# Achieving sustainable performance: synergistic effects of nano-silica and recycled expanded polystyrene in lightweight structural concrete

**DOI:** 10.1038/s41598-024-77029-x

**Published:** 2024-11-04

**Authors:** Sabry A. Ahmed, Esraa Ebrahem, M. S. El-Feky

**Affiliations:** 1 Faculty of Engineering, Zagazig University, Sharkia, Egypt; 2 Department of Civil Engineering, National Research Centre, Cairo, Egypt

**Keywords:** Lightweight concrete, Nano-silica, Expanded polystyrene (EPS), Compressive strength, Flexural strength, Sustainability, Circular economy, Non-structural applications, Water penetration, Engineering, Materials science, Nanoscience and technology

## Abstract

Lightweight concrete, particularly polystyrene concrete, has been extensively utilized in civil engineering for decades. The incorporation of waste expanded polystyrene (EPS) as a filler material in the production of lightweight concrete presents significant advantages from a circular economy perspective. Prior research indicates that increasing the proportion of lightweight aggregates, such as EPS, typically results in reductions in strength and bulk density. The utilization of substantial amounts of EPS waste in the formulation of structural polystyrene concrete is crucial for advancing sustainable construction practices. This study investigates the effects of varying nano-silica content on the bulk density, compressive strength, flexural strength, splitting tensile strength, and water penetration depth of structural polystyrene concrete. Concrete specimens were prepared by substituting 25%, 50%, 75%, and 100% of sand with EPS waste, while evaluating nano-silica contents of 0.75%, 1%, and 1.25%. The findings reveal that increasing the volume fraction of EPS corresponds to a decrease in the concrete’s bulk density. This research provides critical insights into optimizing structural lightweight concrete, thereby promoting advancements in sustainable construction applications.

## Introduction

The construction industry is increasingly focused on developing sustainable and eco-friendly building materials in response to the growing environmental concerns associated with traditional construction practices. Among these materials, lightweight concrete has emerged as a viable alternative, substituting conventional aggregates with lightweight options such as expanded polystyrene (EPS)^1,2^. This innovative material offers numerous advantages, including reduced overall weight, improved thermal insulation, and enhanced acoustic properties, making it suitable for a diverse range of construction applications^1,2^.

However, the incorporation of EPS into concrete presents several challenges. While EPS contributes to reduced density and weight, it can also lead to a significant decrease in mechanical strength and durability of the resulting concrete mixture^3–6^. High EPS content may compromise load-bearing capacity and overall structural integrity, limiting its broader adoption in critical structural applications. Furthermore, the porous nature of EPS can adversely affect the microstructure of concrete, resulting in increased susceptibility to cracking and reduced long-term performance^3–6^. Notably, studies by Islam et al.^7,8,20^ have highlighted the impact of alternative lightweight aggregates on the mechanical behavior and durability of concrete, emphasizing the need for further investigation into the role of EPS in structural applications [70,71].

To mitigate these limitations, researchers have increasingly turned to nanotechnology, specifically the incorporation of nano-silica (SiO_2_), to enhance the performance characteristics of lightweight concrete containing EPS^10–13^. Nano-silica, characterized by its particle size of less than 100 nanometers, exhibits unique properties such as a high specific surface area and reactivity, which enable it to effectively fill voids and cracks within the concrete matrix^10–13^. Various studies have demonstrated that the addition of nano-silica can lead to significant improvements in compressive strength, flexural and tensile strengths, as well as resistance to chloride ion penetration^14–18^. For instance, research conducted by Islam et al. (2022), and Hemn Unis Ahmed et al. (2022) on renewable waste materials underscores the potential for nanomaterials to enhance the mechanical properties of concrete^18,19^.

The enhanced mechanical performance of lightweight concrete with nano-silica can be attributed to several key mechanisms. The large surface area of nano-silica promotes increased reactivity with cementitious materials, fostering a denser and more cohesive concrete matrix. Additionally, the fine particle size allows for effective filling of voids, leading to improved strength and durability. Furthermore, nano-silica particles can serve as nucleation sites during the hydration process, accelerating the formation of hydration products and enhancing overall hydration kinetics^10–13^. A comprehensive review by Islam et al.^7,8,20^ highlights the durability properties of structural lightweight concrete incorporating innovative materials, reinforcing the significance of this research direction^20^.

The primary challenge addressed in this study is the necessity to enhance the performance of lightweight concrete by overcoming the limitations associated with the use of recycled EPS as a substitute for traditional aggregates. While lightweight concrete offers reduced weight, the adverse effects of EPS on strength and durability hinder its application in various construction scenarios, particularly in regions with weak or soft soils that cannot support the loads of multi-story buildings or heavy industrial structures. Enhanced lightweight concrete could provide a viable solution for such contexts, allowing for the construction of stable and resilient structures even on challenging ground conditions.

This research aims to investigate the synergistic effects of nano-silica SiO_2_ and recycled EPS on the mechanical properties and durability of lightweight concrete. Specifically, the objectives include improving compressive strength, flexural strength, and splitting tensile strength, as well as enhancing resistance to harmful ion penetration. By focusing on the interactions between these two materials, this study seeks to develop a more sustainable and high-performance lightweight concrete suitable for a variety of construction applications.

Ultimately, the findings from this research are expected to contribute to the advancement of lightweight concrete technology, addressing existing gaps in knowledge regarding the combined effects of EPS and nano-silica. This work aims to promote the wider application of lightweight concrete in the construction industry, fostering more sustainable and durable building practices. The insights gained could facilitate the development of lightweight concrete solutions that are specifically tailored for use in soft or weak soil conditions, thus expanding the potential for innovative construction methods in diverse environments.

## Materials and methods

This research focuses on the experimental investigation of the synergistic effects of nano-silica (SiO_2_) and recycled expanded polystyrene (EPS) on the performance characteristics of structural lightweight concrete. The scope encompasses a comprehensive analysis of the mechanical properties and durability attributes of lightweight concrete formulated with varying proportions of nano-silica and recycled EPS. Specifically, this study aims to systematically evaluate the impact of different combinations and ratios of nano-silica and recycled EPS on key performance metrics, including compressive strength, flexural strength, and splitting tensile strength. By employing a range of experimental methods, this research seeks to elucidate the mechanisms underlying the interactions between nano-silica and recycled EPS, thereby providing a clearer understanding of their combined effects on the microstructure and overall performance of structural lightweight concrete. The ultimate goal of this research is to optimize the formulation of structural lightweight concrete through the strategic integration of nano-silica and recycled EPS, enhancing its mechanical properties and durability. The findings are expected to contribute significantly to the development of sustainable, high-performance lightweight concrete solutions that can be utilized in a variety of construction applications, particularly in environments where reduced weight and enhanced structural integrity are essential. This research will not only advance the scientific understanding of structural lightweight concrete but also support ongoing efforts towards sustainable construction practices by promoting the use of recycled materials.

### Materials

**Fig. 1:**
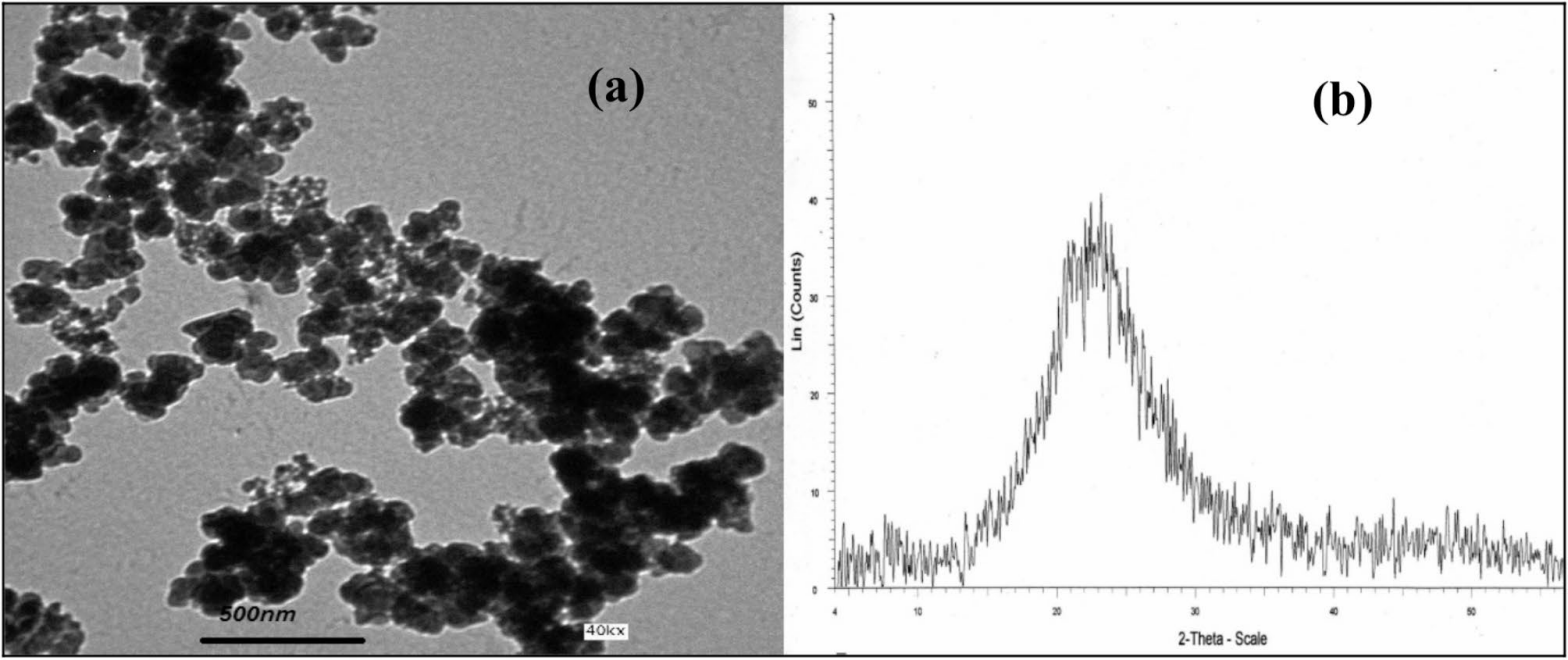
(**a**) TEM micrograph of SiO_2_ Nano particles, (**b**) XRD analysis of SiO_2_ Nano particles.

Ordinary Portland Cement (OPC) conforming to ASTM C150^21^ standard was used as received. Local nano silica was used as a replacement material with 0.75, 1 and 1.25%from cement weight; The properties of SiO_2_ nano silica particles are shown in Table 1. Transmission electron micrographs (TEM) and powder X-ray diffraction (XRD) diagrams of SiO_2_ nano silica particles are shown in Fig. 1 as received by the manufacturer. The expanded polystyrene foam (EPS) was used as a replacement material with 25, 50, 75 and 100 with sand volume; it characteristics are shown in Table 2. It was selected based on its Therefore, it has a very low density (20–30 kg/m^3^).The sand used in mortars is the locally available sand free from alkali-reactive materials. The water used is the potable water from the water-supply network system, free from suspended solid and organic materials, which can affect the properties of the fresh and hardened concrete.

**Table 1:** Chemical composition of Nano SiO_2_ (wt %).

Element	SiO_2_	Fe_2_O_3_	Al_2_O_3_	MgO	CaO	Na_2_O	*P*_2_O_5_
NS	99.17	0.06	0.13	0.11	0.14	0.40	0.01

*NS* Nano Silica (Nano SiO_2_).

**Table 2:** Chemical and physical properties of EPS foam.

Molecular weight	Density (kg/m^3^)	Beads diameter (mm)	Thermal conductivity W/(m.K)	Compressive strength (*N*/mm^2^)
300.000	17	1.5 3	0.04	0.1

### Mixture proportions (experimental method)

As shown in Table 3, a total of 20 mixtures were prepared using a consistent cement to sand ratio of 1:2. A constant water/cement ratio of 0.50 was maintained throughout the experiment.

**Table 3:** Mixing design of EPS foamed concrete with nano silica SiO_2_.

Mix name	Cement (kg)	Sand (cm^3^) |	EPS (cm^3^)	Water (cm^3^)	Nano silica (%)
Control	31.5	35,000	0	15,750	0
N1	31.5	35,000	0	15,750	0.75
N2	31.5	35,000	0	15,750	1
N3	31.5	35,000	0	15,750	1.25
E25	31.5	26,250	8750	15,750	0
E50	31.5	17,500	17,500	15,750	0
E75	31.5	8750	26,250	15,750	0
E100	31.5	0	35,000	15,750	0
E25N0.75	31.5	26,250	8750	15,750	0.75
E25N1	31.5	26,250	8750	15,750	1
E25N1.25	31.5	26,250	8750	15,750	1.25
E50N0.75	31.5	17,500	17,500	15,750	0.75
E50N1	31.5	17,500	17,500	15,750	1
E50N1.25	31.5	17,500	17,500	15,750	1.25
E75N0.75	31.5	8750	26,250	15,750	0.75
E75N1	31.5	8750	26,250	15,750	1
E75N1.25	31.5	8750	26,250	15,750	1.25
E100N0.75	31.5	0	35,000	15,750	0.75
E100N1	31.5	0	35,000	15,750	1
E100N1.25	31.5	o	35,000	15,750	1.25

The control mix, denoted as Mix C, consisted of sand and cement only, without any EPS replacement or nano-silica addition.

To investigate the effect of nano-silica on the mechanical properties of lightweight concrete, three additional mixes, namely N1, N2, and N3, were prepared. These mixes contained the same constituents as Mix C but included nano-silica at 0.75%, 1%, and 1.25% respectively, as a replacement for the weight of cement, the nano silica was added to the mixes as received from the manufacturer.

To study the influence of EPS without nano-silica on the mechanical properties of lightweight concrete, mixes E25, E50, E75, and E100 were prepared. These mixes involved replacing 25%, 50%, 75%, and 100% of the sand volume with EPS.

Furthermore, to examine the combined effects of EPS and nano-silica, additional mixes were prepared. For example, mixes E25N0.75, E25N1, and E25N1.25 contained 25% EPS and 0.75%, 1%, and 1.25% nano-silica, respectively. Similar combinations were prepared for mixes E50N0.75, E50N1, E50N1.25, E75N0.75, E75N1, E75N1.25, E100N0.75, E100N1, and E100N1.25, where the numbers indicate the percentage of EPS and the letters denote the percentage of nano-silica.

The mixing process involved adding the solution to the mixer along with a portion of water and cement, which were mixed for two minutes. The EPS beads were thoroughly mixed with the sand for five minutes. Part of the water was then added to the nano-silica and sprayed during the mixing process to ensure uniform distribution throughout the mixture. Mixing continued until a consistent and homogeneous mixture was achieved.

Test specimens were cast using only hand compaction. The concrete samples were removed from the molds 24 h after casting and placed in a moist curing tank in accordance with ASTM C511^22^ standards.

### Testing

The density of hardened EPS concrete was determined following the guidelines outlined in ASTM C567^23^ at the age of 7 and 28 days.

The compressive strength of hardened EPS lightweight concrete was evaluated using cube specimens measuring 70 × 70 × 70 mm. The tests were conducted at the ages of 7 and 28 days, employing a loading rate of 2.5 kN/s in accordance with ASTM C39^24^.

To analyze the splitting tensile strength behavior, cube specimens measuring 70 × 70 × 70 mm were cast. The splitting tensile strength tests were performed at 28 days of age, following the procedures outlined in ASTM C496^25^.

The flexural strength of the lightweight concrete was assessed using two-point load flexural strength tests conducted according to ASTM C87^26^. Prism-shaped specimens measuring 150 × 150 × 500 mm were used for this test, and the evaluations were performed at the age of 28 days.

The permeability of hardened EPS lightweight concrete was measured using cube specimens measuring 150 × 150 × 150 mm. The permeability tests were conducted to assess the material’s resistance to fluid penetration and followed appropriate procedures in accordance with relevant standards.

All testing procedures were carried out in adherence to the specified standards and guidelines to ensure accurate and comparable results for the evaluation of the mechanical properties and permeability of the hardened EPS lightweight concrete.

### Statistical method

The data collection process involved several steps to ensure quality and accuracy. First, the raw data were obtained from the experimental results. These data were then carefully reviewed and revised to address any inconsistencies, outliers, or missing values. The revised data were then entered into the JMP Pro 13.0.0 statistical software package for further analysis.

The use of JMP Pro 13.0.0 allowed for the application of a robust set of statistical methods to analyze the data. JMP Pro is a powerful data analysis and visualization tool that provides a wide range of statistical techniques, including descriptive statistics, regression analysis, hypothesis testing, and multivariate analysis, among others. The choice of specific statistical methods was guided by the research objectives, the nature of the data, and the underlying assumptions of the analytical techniques.

The data analysis process involved the exploration of the dataset, the identification of relevant variables, the assessment of data distributions and relationships, and the application of appropriate statistical tests to address the research questions. The results of the analyses were then interpreted in the context of the existing literature and the study’s objectives, with due consideration given to the limitations and assumptions of the methods employed.

## Experimental results and discussion

### Density

**Fig. 2:**
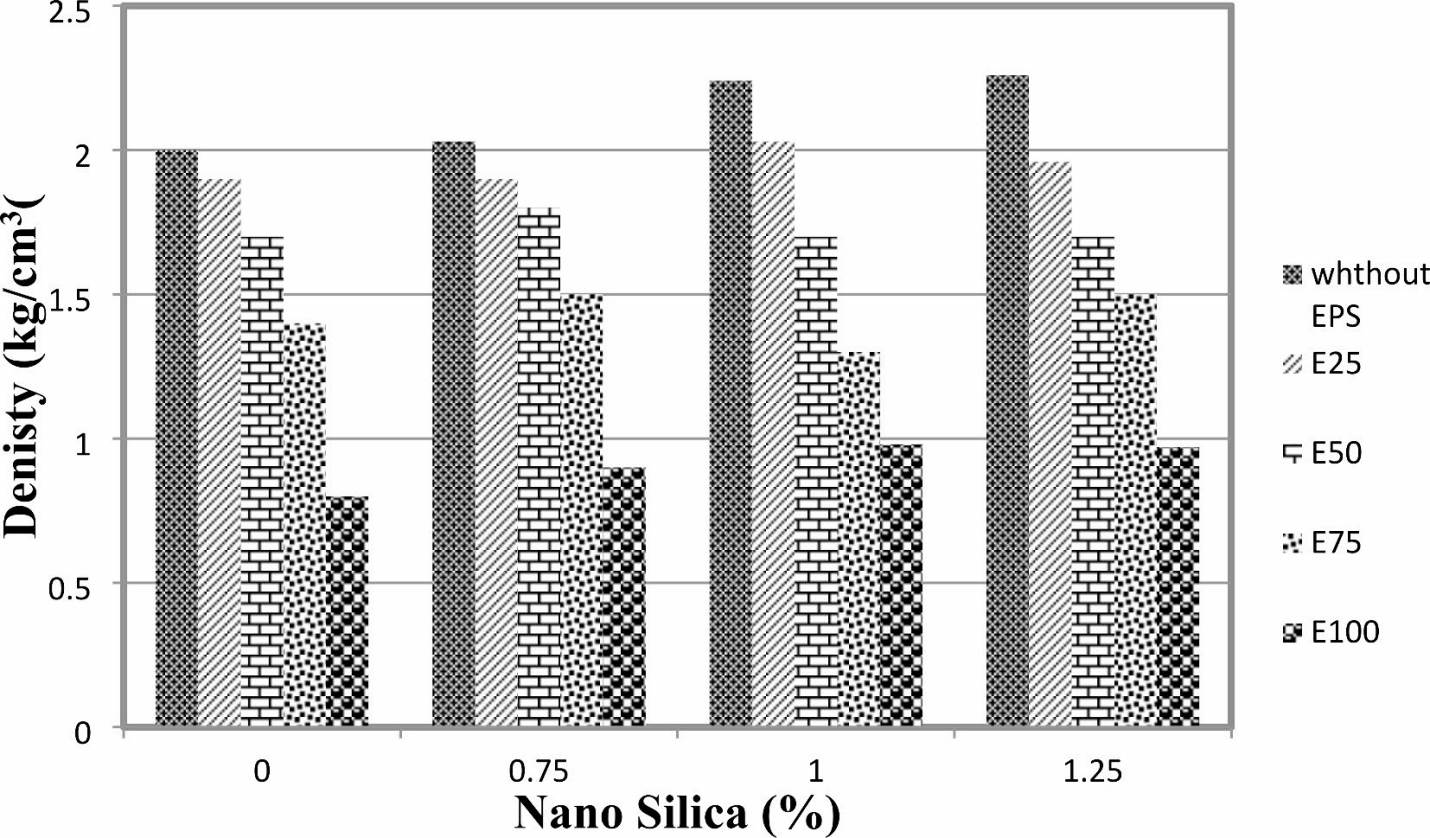
Denisty of mixes with different replacement of EPS with different percentage of NS.

The impact of EPS-C particles and the incorporation of nano-silica on the density of the concrete was investigated. Figure 2 illustrates the variations in concrete density resulting from different percentages of nano-silica addition, both with and without EPS.

When different percentages of nano-silica were used without EPS, the density of the concrete decreased. This observation aligns with the findings of previous studies, which have reported that the addition of nano-silica can lead to a reduction in concrete density. The decrease in density can be attributed to the introduction of nano-silica particles, which possess a lower specific gravity compared to cementitious materials. As a result, the overall weight of the concrete mixture is reduced, leading to a lower density.

On the other hand, when the volume of EPS particles increased, the density of the concrete decreased. This outcome is expected as the incorporation of EPS particles occupies space within the concrete matrix, displacing a portion of the binder material. This displacement results in the formation of macro-pores within the concrete, which contribute to a decrease in overall density.

Interestingly, when different percentages of nano-silica were used in combination with different levels of EPS replacement, the density of the concrete was not significantly affected. This suggests that the use of nano-silica does not have a pronounced impact on the density of lightweight concrete when EPS particles are present. It is worth noting that the combined effect of nano-silica and EPS on the density of concrete warrants further investigation and analysis.

From the results, we observed that as the EPS content increased from 25 to 100%, the bulk density decreased from approximately 2 kg/m^2^ to 0.8 kg/m^2^. This represents a reduction of 60%, indicating a clear inverse relationship between EPS content and bulk density for the control mix, 55% for mixes containing 0.75% nano silica, 56% for mixes containing 1% nano silica, and 57% for the 1.25% nano silica mixes.

In summary, the addition of nano-silica without EPS resulted in a decrease in concrete density, while an increase in the volume of EPS particles led to a reduction in density. The impact of different percentages of nano-silica in combination with various levels of EPS replacement did not significantly influence the density of the lightweight concrete. These findings contribute to our understanding of the factors influencing the density of lightweight concrete and provide valuable insights for optimizing the mixture proportions in future applications.

### Compressive strength

**Fig. 3:**
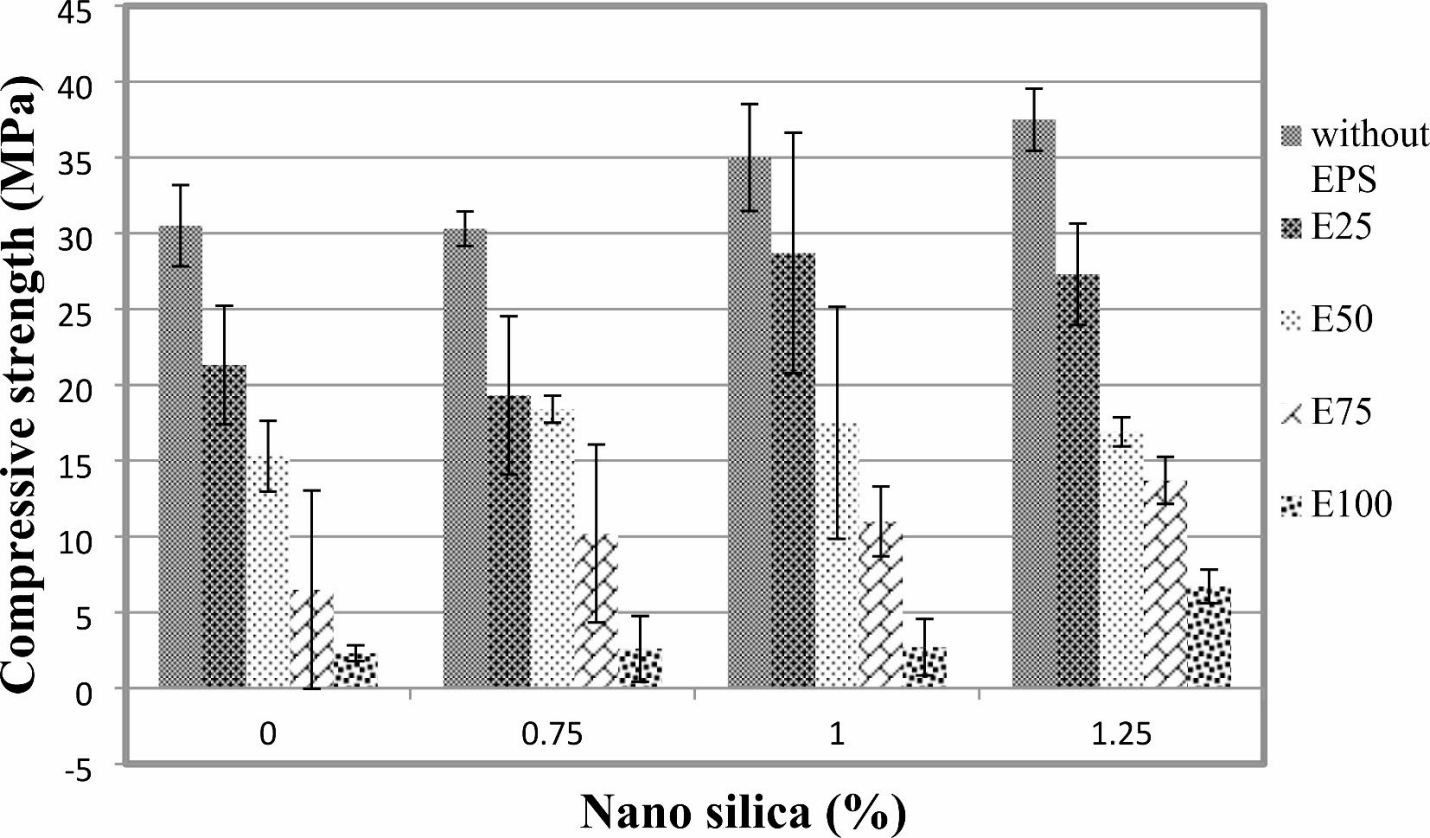
7-day compressive strength of mixes with different replacement of EPS with different percentage of NS.

**Fig. 4:**
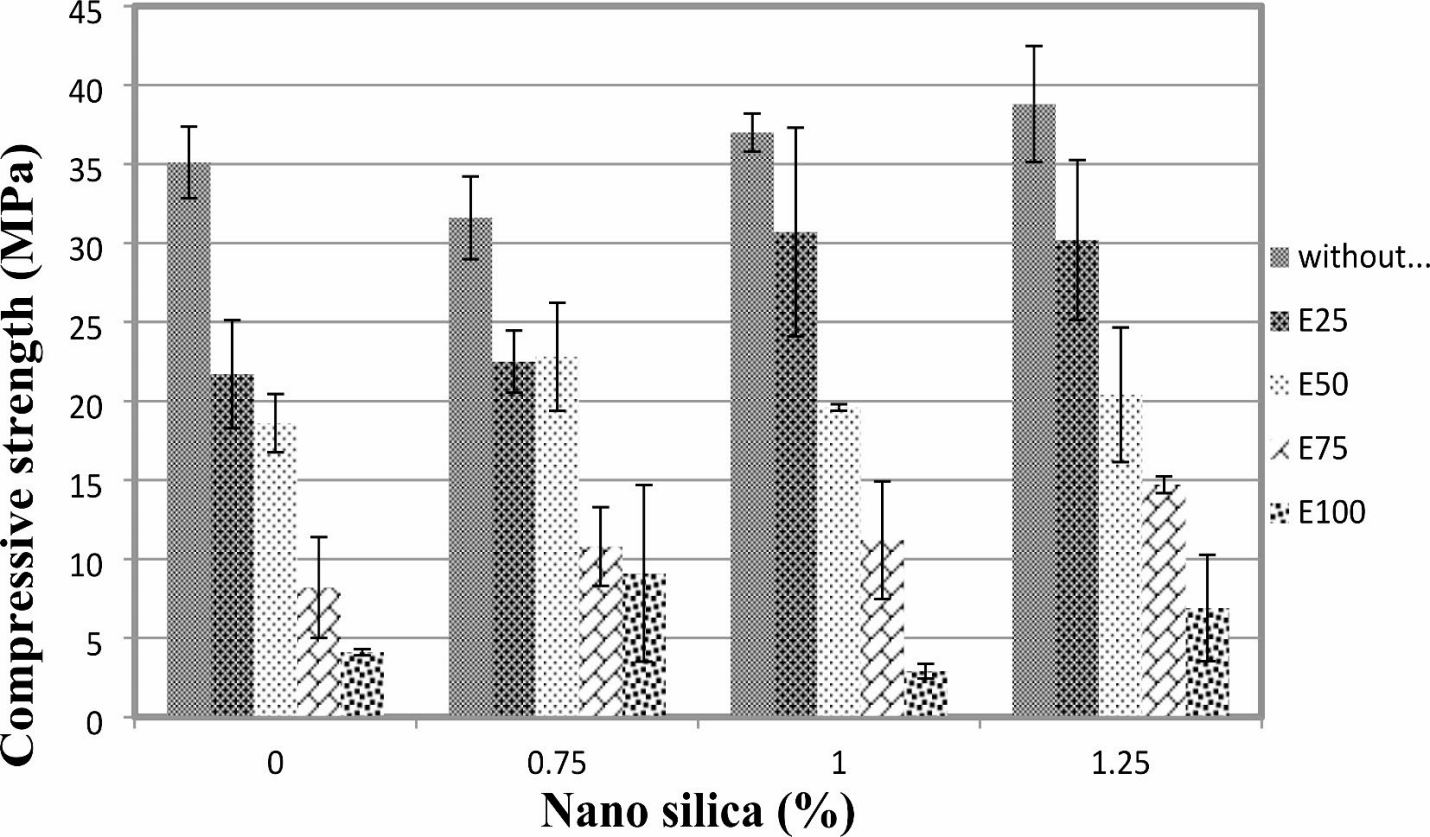
28-day compressive strength of mixes with different replacement of EPS with different percentage of NS.

The compressive strength results at 7 and 28 days are presented in Figs. 3 and 4. It is evident that the addition of nano-silica to the concrete mixture resulted in an increase in compressive strength. At the 7-day age, the inclusion of 1% and 1.25% nano-silica led to a gain in compressive strength of approximately 15% and 36.5%, respectively, compared to the control mix without EPS and nano-silica. However, when 0.75% nano-silica was added, there was a decrease in compressive strength by up to 10%.

At the 28-day age, the compressive strength of the concrete increased by approximately 5% and 10% for mixes with 1% and 1.25% nano-silica, respectively, compared to the control mix. These results indicate that the presence of nano-silica positively influenced the long-term compressive strength of the concrete.

Figure 3 provides a detailed view of the 7-day compressive strengths for mixes with different nano-silica contents. When the mix contained 25% EPS, the inclusion of 0.75% nano-silica resulted in a 9% decrease in compressive strength. However, the presence of 1% and 1.25% nano-silica enhanced the compressive strength by 35% and 28%, respectively, compared to the mix with 25% EPS only. At the 28-day age, the compressive strength showed improvements of approximately 3%, 41%, and 39% for the inclusion of 0.75%, 1%, and 1.25% nano-silica, respectively, compared to the mix with 25% EPS without nano-silica.

A similar trend was observed for the mixes containing 50% EPS, as shown in Figs. 3 and 4. The addition of 0.75%, 1%, and 1.25% nano-silica enhanced the 7-day compressive strength by 20%, 14%, and 10%, respectively, compared to the mix with 50% EPS without nano-silica. At the 28-day age, the compressive strength improvements were approximately 22%, 5%, and 10% for the inclusion of 0.75%, 1%, and 1.25% nano-silica, respectively, compared to the mix with 50% EPS.

For the mixes with 75% EPS, the presence of 0.75%, 1%, and 1.25% nano-silica significantly enhanced the compressive strength at both 7 and 28 days. At 7 days, the compressive strength improvements were 57%, 69%, and 111% for the inclusion of 0.75%, 1%, and 1.25% nano-silica, respectively, compared to the mix with 75% EPS without nano-silica. Similarly, at 28 days, the compressive strength improvements were approximately 32%, 36%, and 79% for the inclusion of 0.75%, 1%, and 1.25% nano-silica, respectively, compared to the mix with 75% EPS.

Lastly, for the mix with 100% EPS, the inclusion of 0.75%, 1%, and 1.25% nano-silica resulted in significant enhancements in compressive strength at 7 days. The improvements were 13%, 17%, and 191%, respectively, compared to the mix with 100% EPS without nano-silica. At 28 days, the compressive strength improvements were approximately 121% and 98% for the inclusion of 0.75% and 1.25% nano-silica, respectively. However, the inclusion of 1% nano-silica resulted in a 29% decrease in 28-day compressive strength compared to the mix with 100% EPS without nano-silica.

The compressive strength results from our study align with previous research indicating that the inclusion of nano-silica can enhance the compressive strength of concrete, particularly in mixtures with varying contents of recycled materials. For instance, studies by Islam et al.^7,8,18,20^ have shown similar trends where nano-silica improves mechanical properties in sustainable concrete formulations. However, our findings also reveal specific instances of strength reduction, such as the 10% decrease in compressive strength with 0.75% nano-silica at 7 days when mixed with 25% EPS. This finding is somewhat unexpected and suggests a complex interaction between nano-silica and EPS that may be influenced by the overall microstructure and bonding characteristics of the concrete matrix. Further investigation into the optimal ratios of nano-silica and EPS, as well as their interaction mechanisms, is warranted to elucidate these effects and optimize lightweight concrete formulations for enhanced performance. However, these findings highlight the positive influence of nano-silica on the compressive strength of lightweight concrete, with the magnitude of improvement depending on the content of nano-silica and the percentage of EPS replacement. The results provide valuable insights for optimizing the mixture proportions and understanding the mechanical behavior of EPS lightweight concrete.

### Flexural strength

**Fig. 5:**
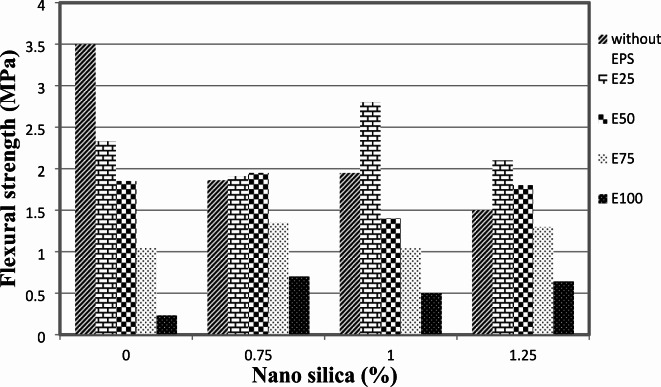
Flexural for mixes of mixes with different replacement of EPS with different percentage of NS.

The flexural strength results at 28 days for mixes without EPS are depicted in Fig. 5. It is evident from the observations that the addition of nano-silica (NS) to the concrete mixture led to a decrease in flexural strength. The reduction in flexural strength was approximately 47%, 44%, and 57% for mixes containing 0.75%, 1%, and 1.25% nano-silica, respectively, compared to the control mix without NS and EPS.

For mixes with 25% EPS, Fig. 5 illustrates the 28-day flexural strength for different contents of nano-silica. In general, the presence of nano-silica had a negative effect on the 28-day flexural strength of cement mortar. The flexural strength decreased by approximately 18% and 10% with the use of 0.75% and 1.25% nano-silica, respectively. However, the flexural strength increased by about 20% with the inclusion of 1% nano-silica compared to the mix with EPS only.

Similarly, for mixes with 50% EPS, Fig. 5 shows the 28-day flexural strength for different contents of nano-silica. The presence of nano-silica also had a negative impact on the 28-day flexural strength of cement mortar in this case. The flexural strength decreased by approximately 24% and 3% with the use of 1% and 1.25% nano-silica, respectively. However, there was a slight increase of about 5% in flexural strength with the inclusion of 0.75% nano-silica compared to the mix with EPS only.

The increase in flexural strength with 1% nano-silica in the 25% EPS mix can be attributed to improved bonding and reduced voids within a less porous matrix, allowing for enhanced load distribution. In contrast, when the EPS content is increased to 50%, the concrete matrix becomes more porous and heterogeneous, which may diminish the effectiveness of nano-silica in enhancing flexural strength. The presence of higher EPS may disrupt the uniform distribution of nano-silica, leading to a less effective interaction and resulting in a decrease in flexural strength. This highlights the importance of carefully balancing EPS content and nano-silica dosage to achieve the desired mechanical properties.

In contrast, for mixes with 75% EPS, Fig. 5 demonstrates that the presence of nano-silica increased the 28-day flexural strength of cement mortar. The flexural strength improved by around 28% and 24% with the use of 0.75% and 1.25% nano-silica, respectively. However, there was a slight decrease of about 0.6% in flexural strength with the inclusion of 1% nano-silica compared to the mix with EPS only.

Lastly, for mixes with 100% EPS, Fig. 5 shows the 28-day flexural strength for different contents of nano-silica. In general, the presence of nano-silica resulted in an increase in the 28-day flexural strength of cement mortar. The flexural strength improved by approximately 204%, 117%, and 178% with the use of 0.75%, 1%, and 1.25% nano-silica, respectively, compared to the mix with EPS only.

These findings indicate that the effect of nano-silica on flexural strength is highly dependent on the EPS content in the concrete mixture. While the addition of nano-silica generally led to a decrease in flexural strength for mixes without EPS, the impact varied for mixes with different EPS contents. It is important to carefully consider the desired flexural strength requirements when incorporating nano-silica into the concrete mixture.

### Splitting tensile strength

**Fig. 6:**
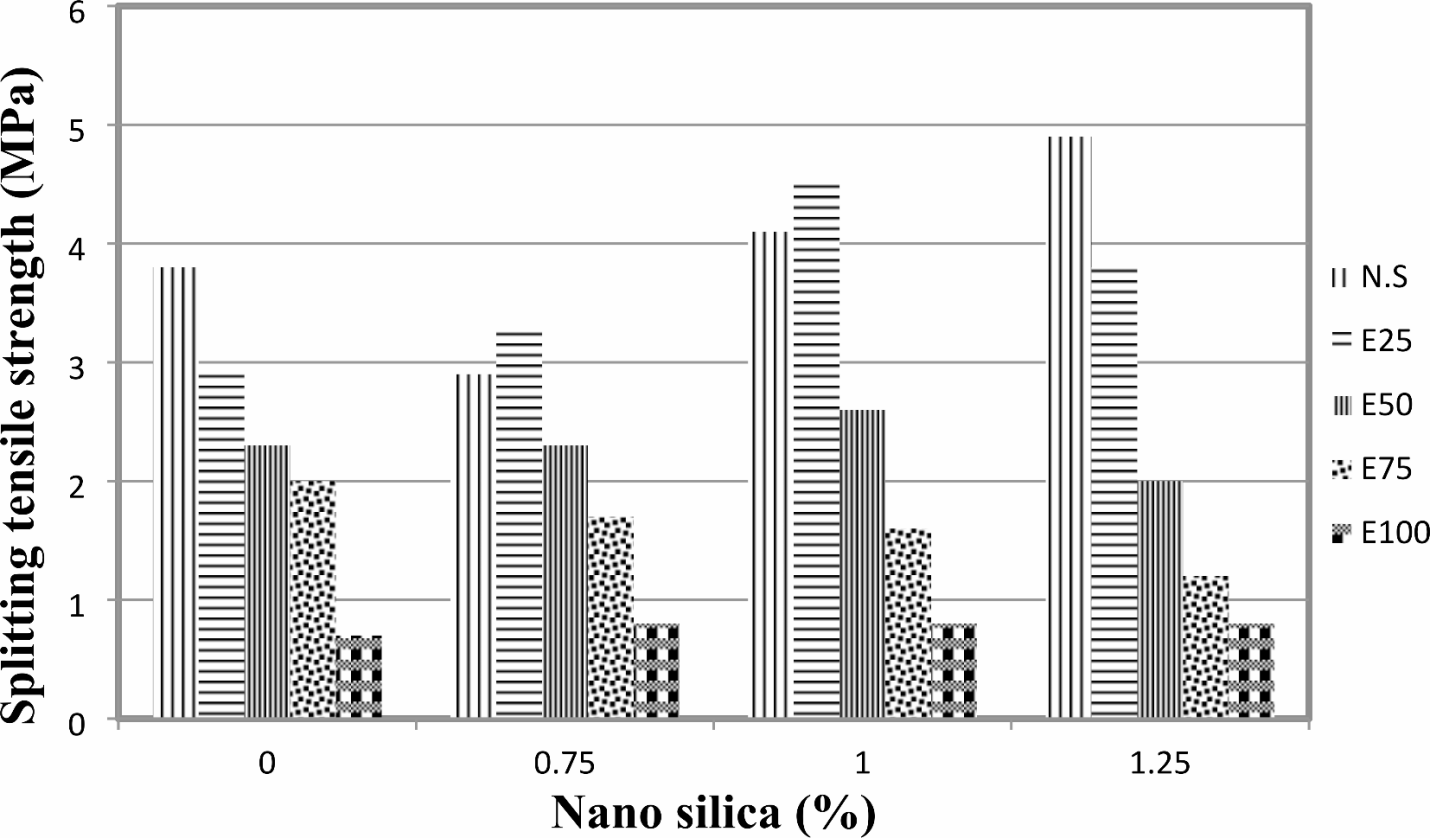
Splitting tensile strength for mixes with NS without EPS.

The splitting tensile strength results at 28 days for mixes without EPS are presented in Fig. 6. It is evident from the observations that adding 0.75% nano-silica (NS) to the mix constituents resulted in a decrease in the splitting strength of concrete. The loss in splitting strength was approximately 24%. However, for mixes with 1% and 1.25% NS, there was an increase in splitting strength of about 8% and 29%, respectively, compared to the control mix without NS and EPS. For mixes with 25% EPS, Fig. 6 demonstrates the 28-day splitting tensile strength at different contents of NS. In general, the presence of NS showed an increase in the 28-day splitting tensile strength. The splitting strength improved by approximately 14%, 55%, and 31% with the use of 0.75%, 1%, and 1.25% NS, respectively, compared to the mix with EPS only. Similarly, for mixes with 50% EPS, the presence of NS showed an increase in the 28-day splitting tensile strength by about 13% with the use of 1% NS. However, 0.75% NS did not affect the splitting strength, while using 1.25% NS decreased the splitting strength by about 13% compared to the mix with EPS only.

In contrast, for mixes with 75% EPS, the presence of NS had a negative effect on the 28-day splitting tensile strength. The splitting strength decreased by approximately 15%, 20%, and 40% with the use of 0.75%, 1%, and 1.25% NS, respectively, compared to the mix with EPS only.

For mixes with 100% EPS, the presence of NS showed an increase in the 28-day splitting strength. The splitting strength increased by about 14%, 14%, and 14% with the use of 0.75%, 1%, and 1.25% NS, respectively, compared to the mix with EPS only.

These findings indicate that the effect of NS on splitting tensile strength is influenced by the presence of EPS in the concrete mixture. The impact of NS on splitting strength can vary depending on the NS content and the amount of EPS used. It is important to consider these factors when designing concrete mixtures to achieve the desired splitting tensile strength.

### Water permeability

**Fig. 7:**
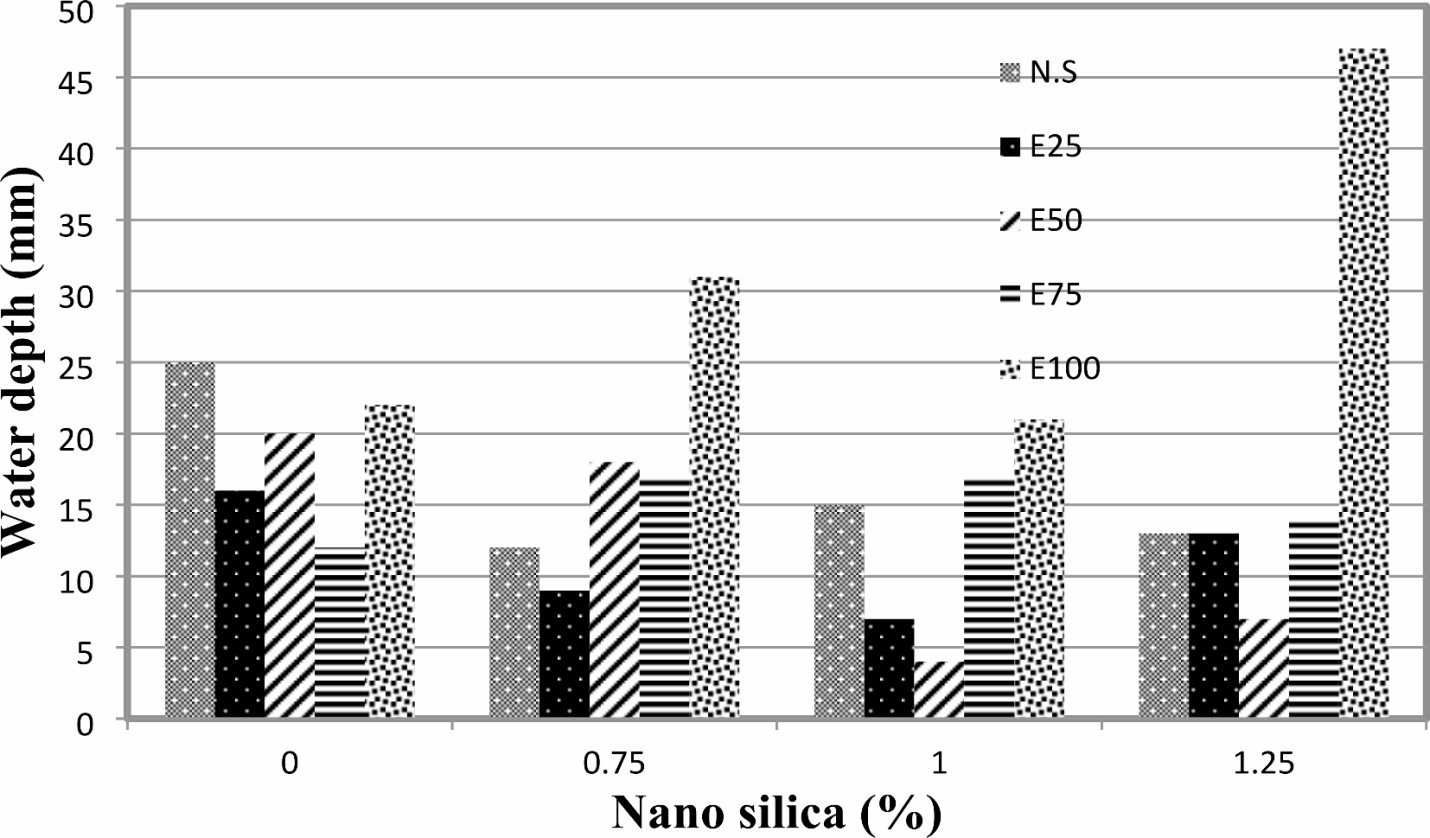
Water permeability for mixes with NS without EPS.

Water permeability of EPS-C is influenced by various factors including cement content, concrete density, aggregate characteristics, and mineral admixtures. The experimental results on the relationship between water permeability and the percentage of nano-silica are depicted in Fig. 7. It can be observed that the use of 0.75%, 1%, and 1.25% of nano-silica resulted in a decrease in water permeability by approximately 52%, 40%, and 48%, respectively.

The relationship between water permeability, nano-silica percentage, and EPS is also presented in Fig. 7. Compared to the mix with 25% EPS replacement, the use of nano-silica at 0.75%, 1%, and 1.25% decreased water permeability by about 44%, 56%, and 19%, respectively. Similarly, compared to the mix with 50% EPS replacement, the use of nano-silica at 0.75%, 1%, and 1.25% decreased water permeability by approximately 10%, 80%, and 65%, respectively. However, for mixes with 75% EPS replacement, the presence of nano-silica had contrasting effects on water permeability. The use of 0.75% nano-silica decreased water permeability by about 42%, while the use of 1% and 1.25% nano-silica increased water permeability by 42% and 17%, respectively.

Finally, for mixes with 100% EPS replacement, the presence of nano-silica resulted in an increase in water permeability. The use of 0.75%, 1%, and 1.25% nano-silica increased water permeability by approximately 158%, 75%, and 291%, respectively, compared to the mix with EPS only. The increase in permeability observed in high EPS content mixes with nano-silica can be attributed to several interrelated factors. Although nano-silica generally enhances the density and reduces the permeability of concrete, the high volume of EPS can create a network of interconnected voids that may not be effectively filled by the nano-silica particles. This void network can facilitate water ingress, leading to increased permeability. Additionally, the incorporation of EPS may introduce micro-cracking or weak points in the matrix, which can further contribute to pathways for water movement. These findings highlight the complex relationship between nano-silica content, EPS replacement, and water permeability in EPS-C. The inclusion of nano-silica can have varying effects on water permeability depending on the EPS content and the percentage of nano-silica used. It is crucial to consider these factors when assessing the water permeability characteristics of EPS-C in practical applications.

## Evaluation of the results

The following are the outcomes of the statistical analysis, which examined the compressive strength, tensile strength, and flexural strength, density and penetration depth of the mixes, considering various ratios of EPS and nano Silica (Ns):

### Conditional sums of squares and analysis of variance

The analysis of variance Tables 4, 5, 6, 7, 8, 9, 10, 11, 12, 13, 14, 15, 16, 17, 18, 19, 20 and 21 breaks down the total observed variability in the dependent variables (compressive strength, tensile strength, and flexural strength, density and penetration depth). It decomposes the variability into two components: one attributed to the regression model and the other due to deviations around the fitted model. The R-squared statistic, calculated by dividing the model sum of squares by the total (corrected) sum of squares, indicates that the model explains [98%, 97%, 93%, 92%, 99%, and 84%] and [96%, 93%, 84%, 81%, 97%, and 63%] strength at 7 days and 28 days, tensile strength, and flexural strength, density and penetration depth, respectively.

**Table 4:** 7-day compressive strength summary of fit.

Response	Value
RSquare	0.983525
RSquare Adj	0.962932
Root mean square error	2.320894
Mean of response	17.89
Observations (or Sum Wgts)	10

**Table 5:** 28-day compressive strength summary of fit.

Response	Value
RSquare	0.970503
RSquare Adj	0.933632
Root mean square error	3.056224
Mean of response	21.02
Observations (or Sum Wgts)	10

**Table 6:** Flexural strength summary of fit.

Response	Value
RSquare	0.932777
RSquare Adj	0.848749
Root mean square error	0.358406
Mean of response	1.598
Observations (or Sum Wgts)	10

**Table 7:** Spilitting tensile strength summary of fit.

Response	Value
RSquare	0.916843
RSquare Adj	0.812897
Root mean square error	0.576757
Mean of response	2.27
Observations (or Sum Wgts)	10

**Table 8:** Denisty summary of fit.

Response	Value
RSquare	0.986948
RSquare Adj	0.970632
Root mean square error	0.088629
Mean of response	1.596
Observations (or Sum Wgts)	10

**Table 9:** Water depth summary of fit.

Response	Value
RSquare	0.839289
RSquare Adj	0.638401
Root mean square error	6.803579
Mean of response	21.3
Observations (or Sum Wgts)	10

**Table 10:** 7-day compressive strength analysis of Variance.

F Ratio	Mean square	Sum of squares	DF	Source
18.0536	257.257	1286.2828	5	Model
**Prob > F**	5.387	21.5462	4	Error
< 0.0001		1307.8290	9	C. Total

**Table 11:** 28-day compressive strength analysis of Variance.

F Ratio	Mean square	Sum of squares	DF	Source
26.3214	245.855	1229.2740	5	Model
Prob > F	9.341	37.3620	4	Error
< 0.0001		1266.6360	9	C. Total

**Table 12:** Flexural strength analysis of Variance.

F Ratio	Mean square	Sum of squares	DF	Source
11.1008	1.42595	7.1297398	5	Model
Prob > F	0.12846	0.5138202	4	Error
< 0.0001		7.6435600	9	C. Total

**Table 13:** Spilitting tensile strength analysis of Variance.

F Ratio	Mean square	Sum of squares	DF	Source
8.8204	2.93408	14.670405	5	Model
Prob > F	0.33265	1.330595	4	Error
< 0.0001		16.001000	9	C. Total

**Table 14:** Denisty Analysis of Variance.

F Ratio	Mean square	Sum of squares	DF	Source
60.4915	0.475164	2.3758198	5	Model
Prob > F	0.007855	0.0314202	4	Error
< 0.0001		2.4072400	9	C. Total

**Table 15:** Water depth analysis of Variance.

F Ratio	Mean square	Sum of squares	DF	Source
4.1779	193.389	966.9452	5	Model
**Prob > F**	\49.289	185.1548	4	Error
0.0954		1152.1000	9	C. Total

**Table 16:** 7-day compressive strength parameters estimate. The *symbol in the tables represents the most significant studied factor.

Prob>|t|	t Ratio	Std error	Estimate	Term
0.0003*	12.22	1.386999	16.942857	Intercept
0.0842	2.29	0.947501	2.1666667	NANOSILICA
0.0001*	-15.25	0.947501	-14.45	EPS
0.6053	-0.56	1.160447	-0.65	NANOSILICA*EPS
0.7067	0.40	1.519382	0.6142857	NANOSILICA*NANOSILICA
0.5601	0.63	1.519382	0.9642857	EPS*EPS

**Table 17:** 28-day compressive strength parameters estimate. The *symbol in the tables represents the most significant studied factor.

Prob>|t|	t Ratio	Std error	Estimate	Term
0.0003*	11.83	1.826443	21.607143	Intercept
0.3297	1.11	1.247698	1.3833333	NANOSILICA
0.0003*	-11.41	1.247698	-14.23333	EPS
0.8901	-0.15	1.528112	-0.225	NANOSILICA*EPS
0.6714	-0.46	2.000768	-0.914286	NANOSILICA*NANOSILICA
0.9759	-0.03	2.000768	-0.064286	EPS*EPS

**Table 18:** Flexural strength parameters estimate. The *symbol in the tables represents the most significant studied factor.

Prob>|t|	t Ratio	Std error	Estimate	Term
0.0010*	8.69	0.214189	1.8607143	Intercept
0.1351	-1.87	0.146319	-0.273333	NANOSILICA
0.0038*	-6.03	0.146319	-0.881667	EPS
0.0282*	3.36	0.179203	0.6025	NANOSILICA*EPS
0.8306	0.23	0.234632	0.0535714	NANOSILICA*NANOSILICA
0.1043	-2.09	0.234632	-0.491429	EPS*EPS

**Table 19:** Spilitting tensile strength parameters estimate. The *symbol in the tables represents the most significant studied factor.

Prob>|t|	t Ratio	Std error	Estimate	Term
0.0039*	5.99	0.344678	2.0642857	Intercept
0.6015	0.57	0.23546	0.1333333	NANOSILICA
0.0029*	-6.51	0.23546	-1.533333	EPS
0.4788	-0.78	0.288379	-0.225	NANOSILICA*EPS
0.4426	0.85	0.377576	0.3214286	NANOSILICA*NANOSILICA
0.9575	0.06	0.377576	0.0214286	EPS*EPS

**Table 20:** Denisty parameters estimate. The *symbol in the tables represents the most significant studied factor.

Prob>|t|	t Ratio	Std error	Estimate	Term
< 0.0001*	33.22	0.052966	1.7592857	Intercept
0.1187	1.98	0.036183	0.0716667	NANOSILICA
< 0.0001*	-16.67	0.036183	-0.603333	EPS
0.6384	-0.51	0.044314	-0.0225	NANOSILICA*EPS
0.7649	-0.32	0.058021	-0.018571	NANOSILICA*NANOSILICA
0.0120*	-4.37	0.058021	-0.253571	EPS*EPS

**Table 21:** Water depth parameters estimate. The *symbol in the tables represents the most significant studied factor.

Prob>|t|	t Ratio	Std error	Estimate	Term
0.0201*	3.74	4.065916	15.214286	Intercept
1.0000	0.00	2.77755	0	NANOSILICA
0.0399*	3.00	2.77755	8.3333333	EPS
0.0530	2.72	3.40179	9.25	NANOSILICA*EPS
0.8217	0.24	4.453988	1.0714286	NANOSILICA*NANOSILICA
0.1114	2.04	4.453988	9.0714286	EPS*EPS

The mean squared error estimates the variance of the deviations around the fitted model. The estimated values are [2.3209, 3.0562, 0.3584, 0.5768, 0.0886, and 6.8036] for compressive strength at 7 days and 28 days, tensile strength, and flexural strength, density and penetration depth, respectively.

The statistical significance of the model is determined by the P value corresponding to the F-ratio. For compressive strength at 7 days and 28 days, and tensile strength, flexural strength, density, the P values are 0.0012, 0.0037, and 0.0185, 0.0278, 0.0007, respectively, indicating that the model is statistically significant. However, for the penetration depth, the P value is 0.0954, suggesting that the model is not statistically significant based on 95% confidence level.

These statistical findings provide valuable insights into the significance and effectiveness of the model in explaining the variations in compressive strength, tensile strength, and flexural strength, density and penetration depth, respectively for different ratios of EPS and NS.

**Fig. 8:**
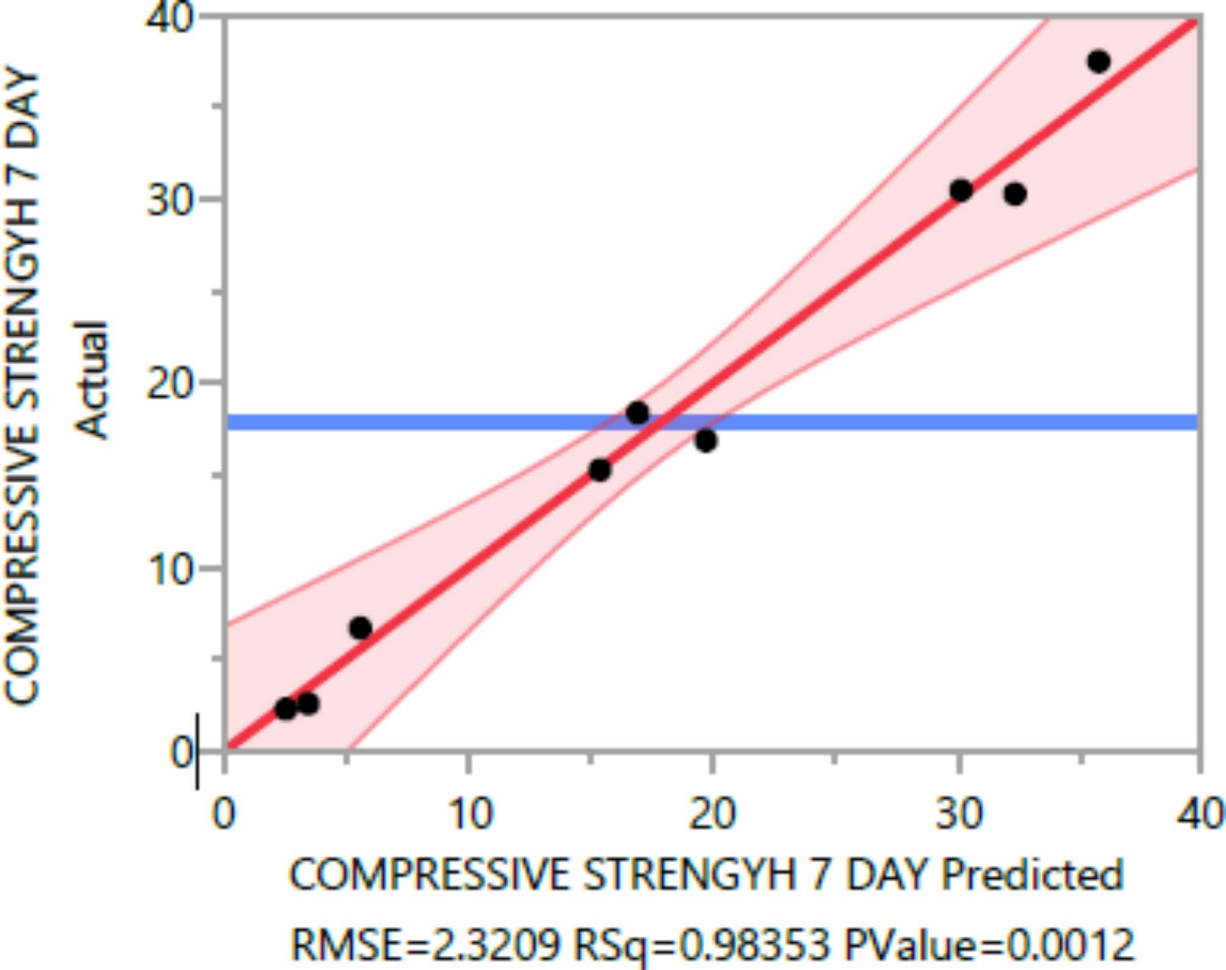
7-dayCompressive Response strength Whole Model Actual by Predicted Plot.

**Fig. 9:**
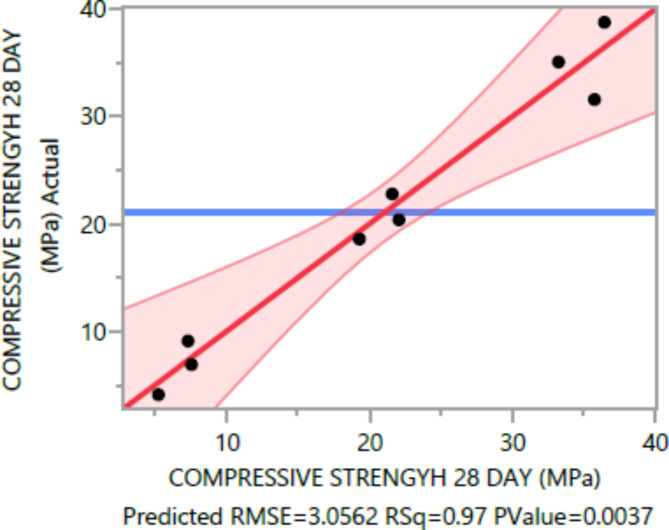
28-dayCompressive Response strength Whole Model Actual by Predicted Plot.

**Fig. 10:**
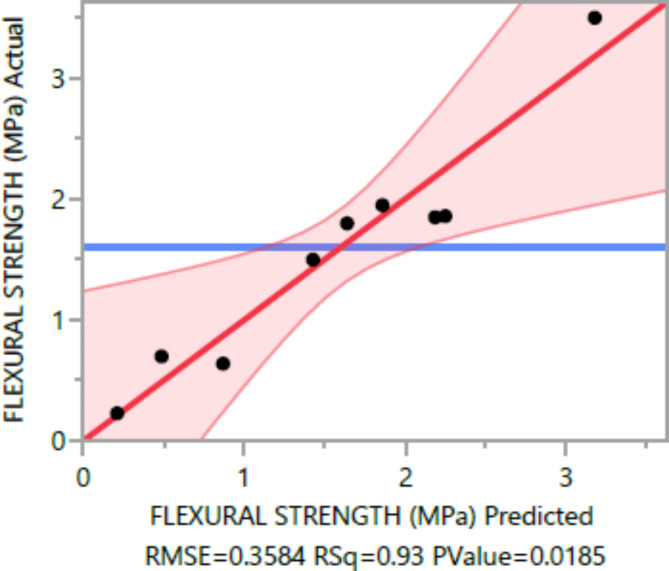
Flexural Response strength Whole Model Actual by Predicted Plot.

**Fig. 11:**
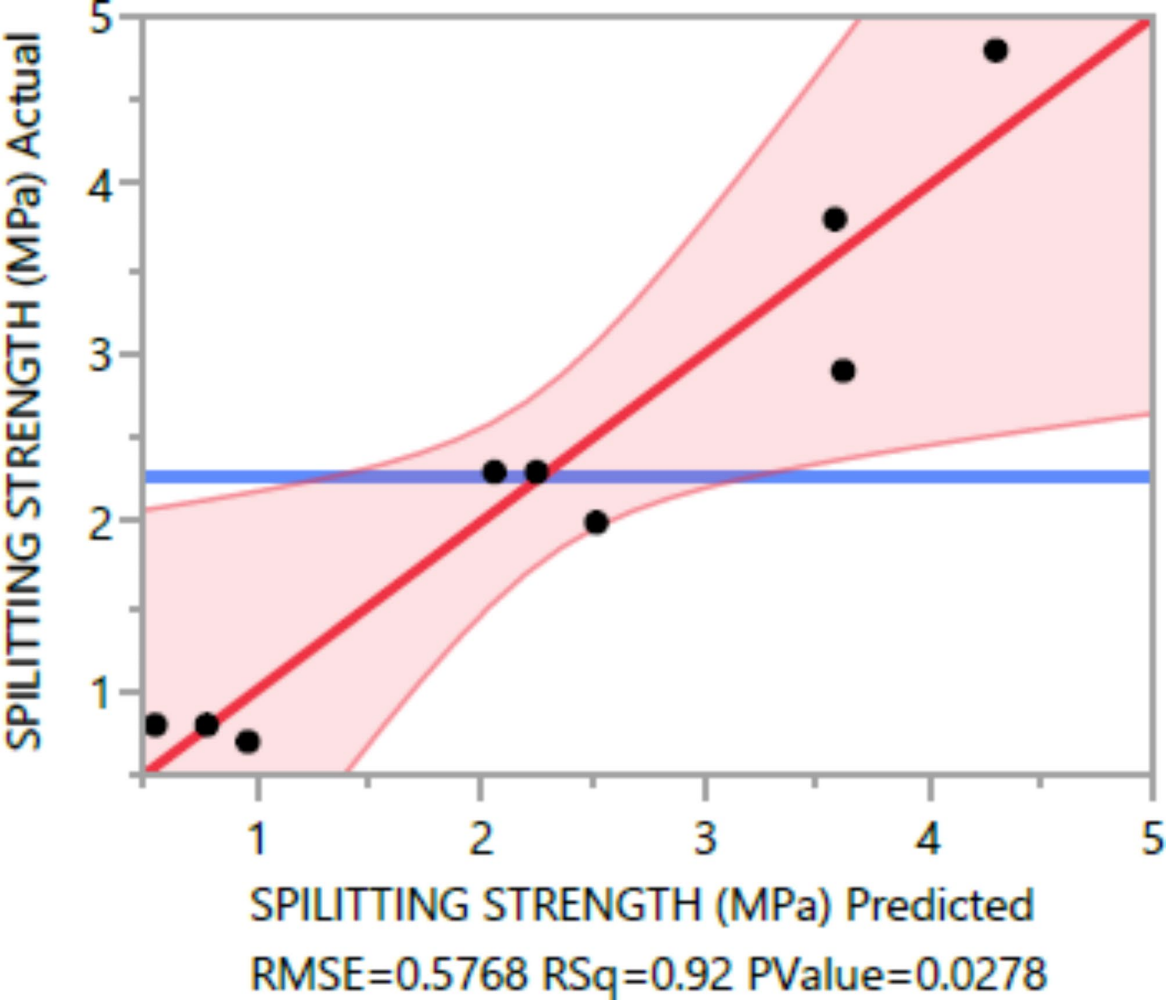
Spilitting Response strength Whole Model Actual by Predicted Plot.

**Fig. 12:**
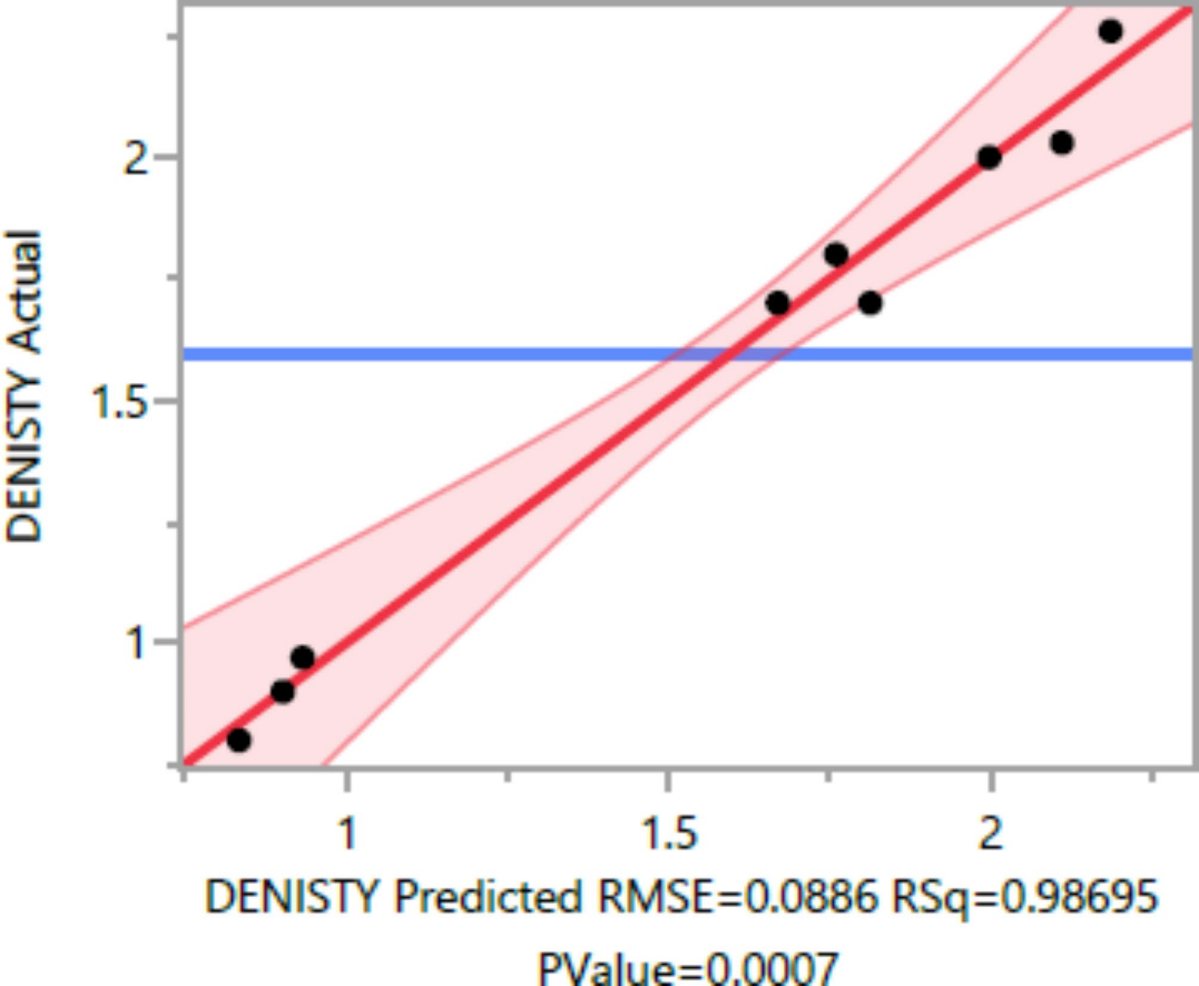
Denisty Whole Model Actual by Predicted Plot.

**Fig. 13:**
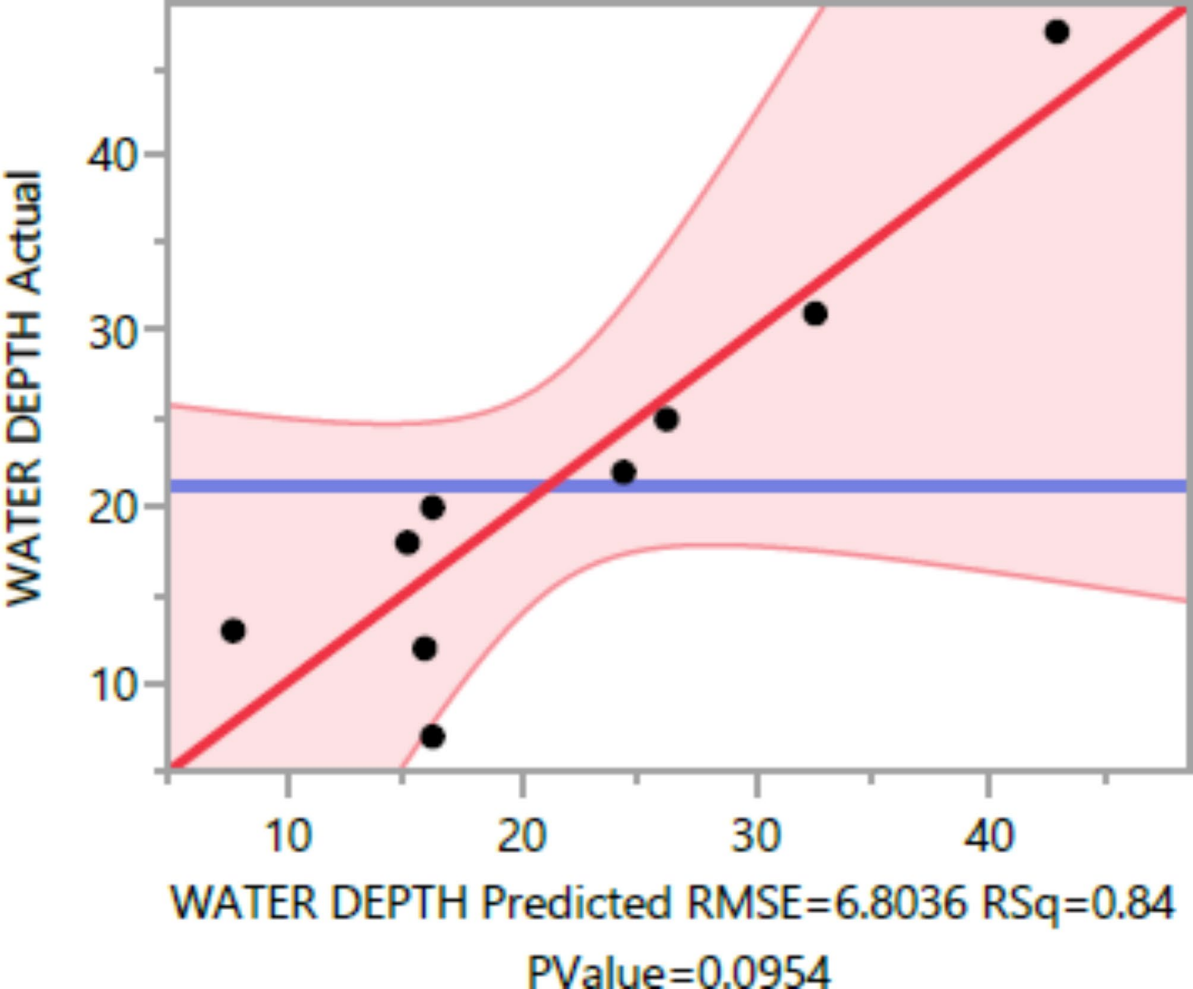
Water depth Whole Model Actual by Predicted Plot.

**Fig. 14:**
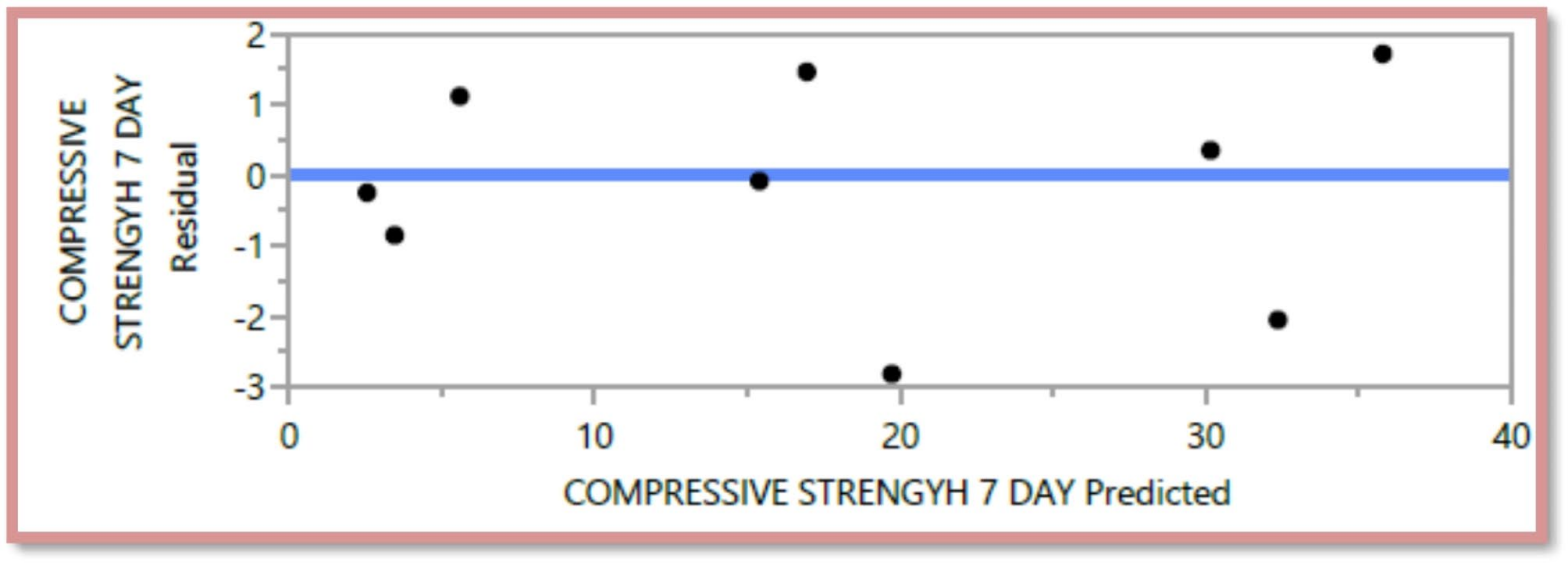
7-day compressive Response strength Whole Model predicted by residual Plot.

**Fig. 15:**
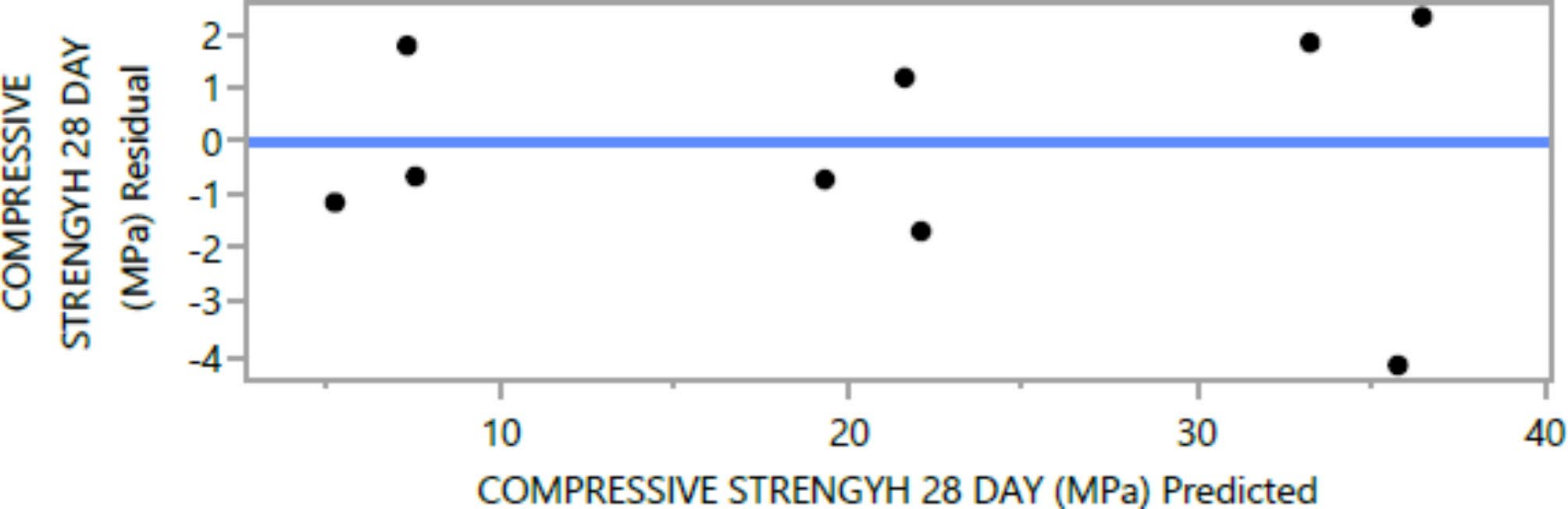
28-day compressive Response strength Whole Model predicted by residual Plot.

**Fig. 16:**
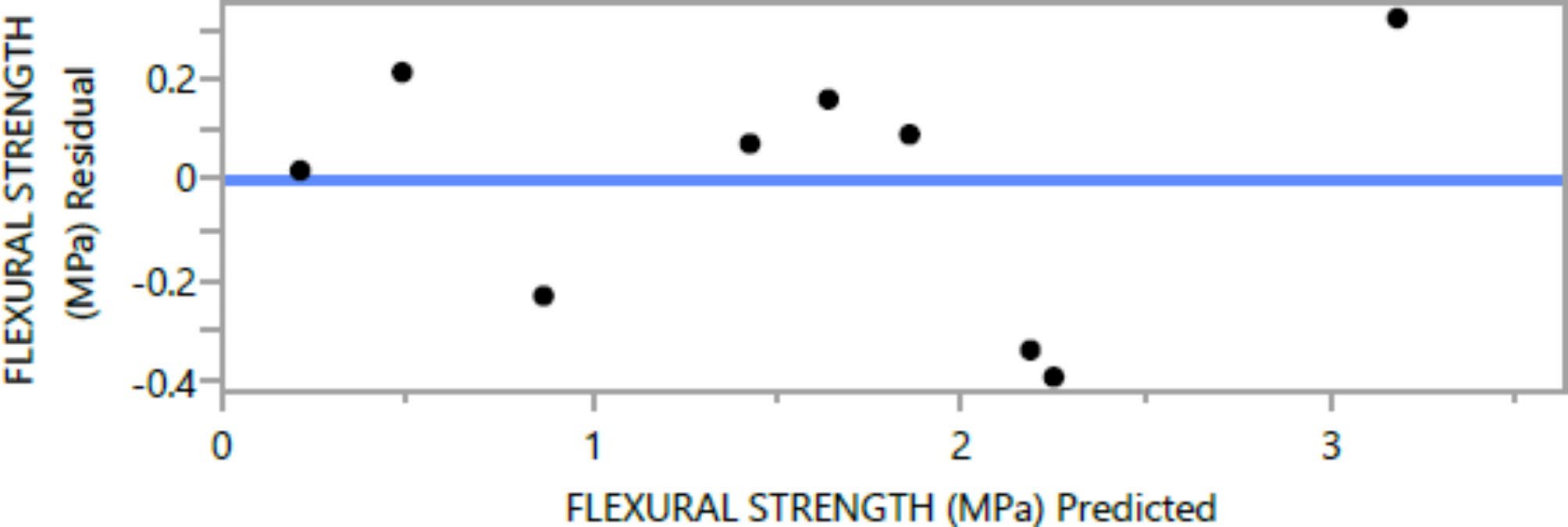
Flexural Response strength Whole Model predicted by residual Plot.

**Fig. 17:**
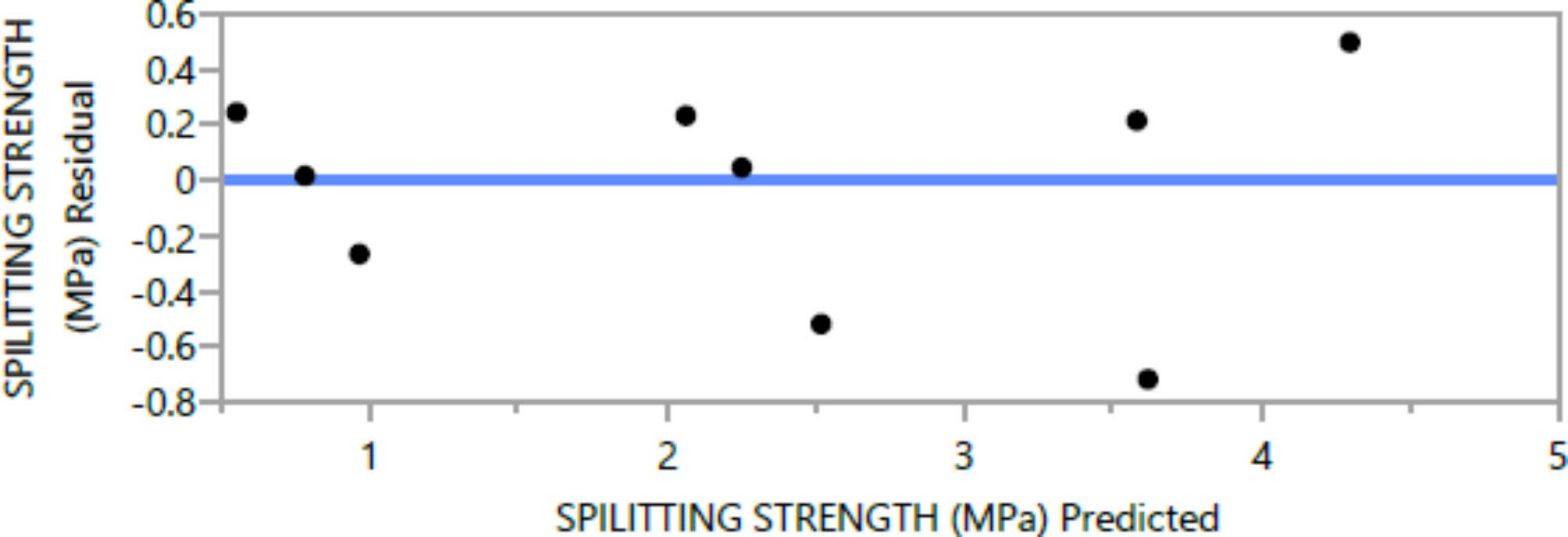
Spilitting Response strength Whole Model predicted by residual Plot.

**Fig. 18:**
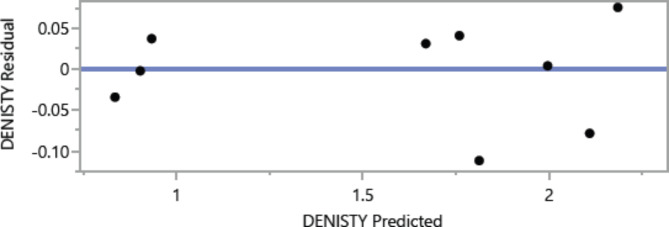
Denisty Whole Model predicted by residual Plot.

**Fig. 19:**
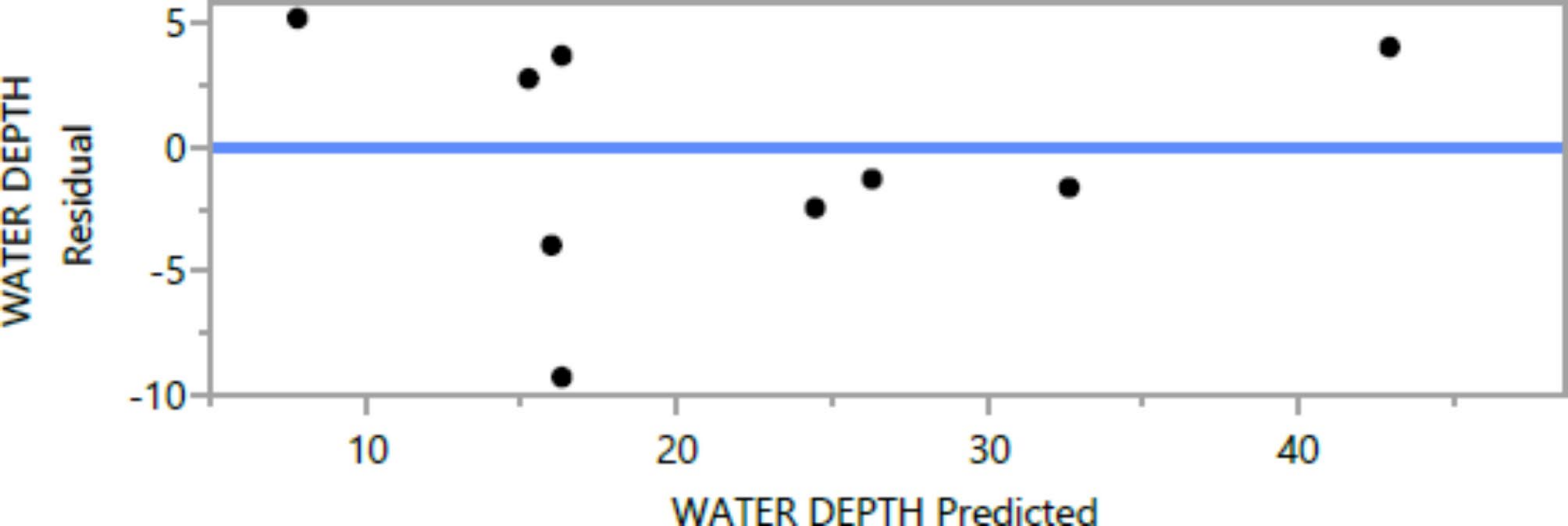
Water depth Whole Model Actual by residual Plot.

Figures 8, 9, 10, 11, 12, 13, 14, 15, 16, 17, 18 and 19 present graphical representations of the standardized residuals in relation to the predicted values for various parameters such as compressive strength at 7 days and 28 days, as well as tensile strength, flexural strength, density, and penetration depth. These residuals refle residuals reflect the disparities between the actual values and the corresponding predictions. Standardized residuals are employed to quantify each deviation in terms of the number of standard deviations away from the fitted line. These residuals were calculated based on the estimated residual standard deviation, assuming that the fitting process did not include the data point in question. This type of residual is particularly useful for identifying outliers, which are data points that deviate significantly from the overall pattern observed in the dataset.

Upon careful examination of Figs. 8, 9, 10, 11, 12, 13, 14, 15, 16, 17, 18 and 19, it becomes evident that the plot demonstrates a satisfactory level of randomness, with no noticeable outliers observed among the residuals. The normal probability plot of the residuals, as depicted in Figs. 8, 9, 10, 11, 12, 13, 14, 15, 16, 17, 18 and 19, provides further insights into whether the residuals can be reasonably assumed to follow a normal distribution. Additionally, this plot aids in the detection of outliers. The residuals align reasonably well along a straight line, indicating adherence to the assumption of a normal distribution. Furthermore, no outliers are discernible in the plot.

**Fig. 20:**
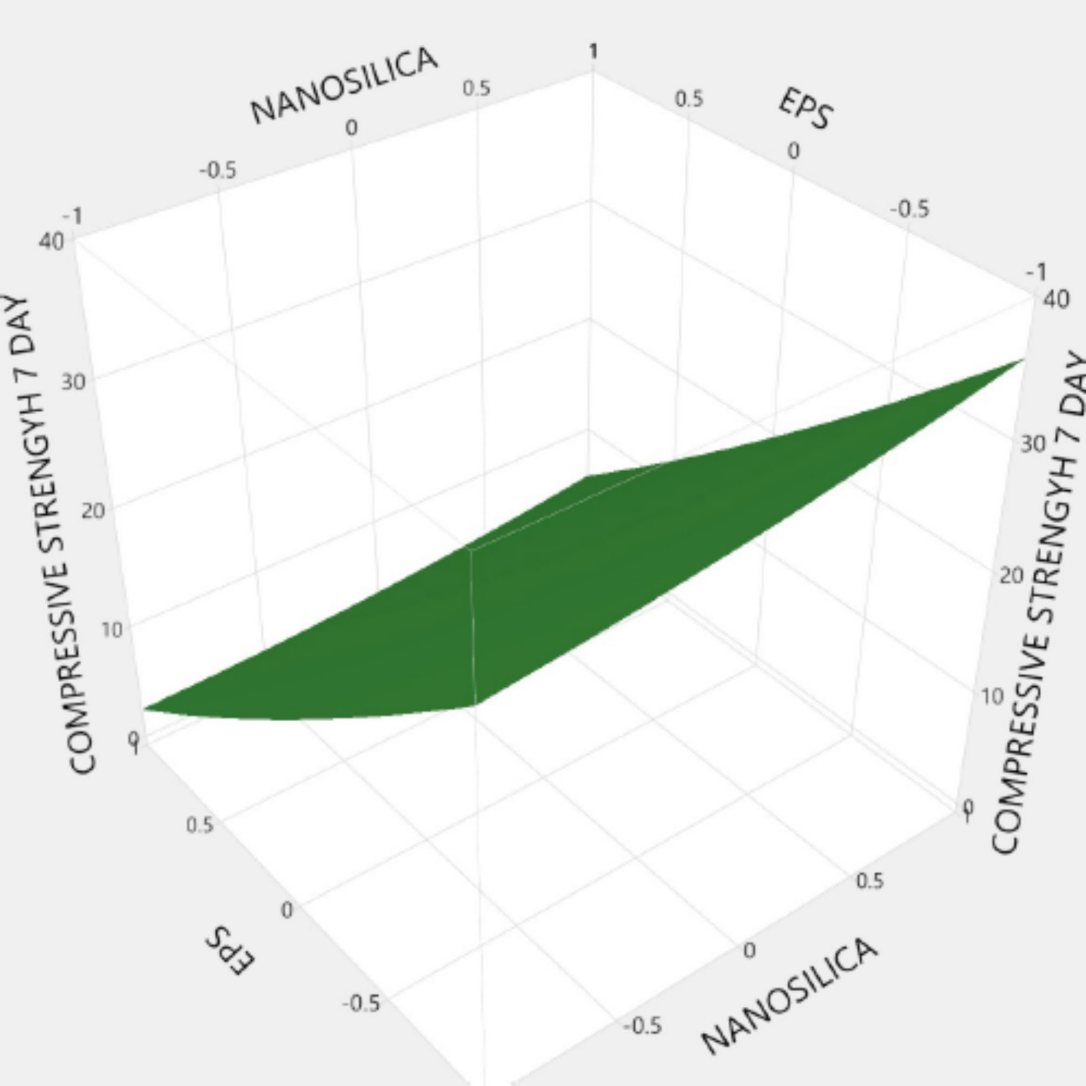
Surface Response of 7-day Compressive strength with the change, NS, and EPS percentages.

**Fig. 21:**
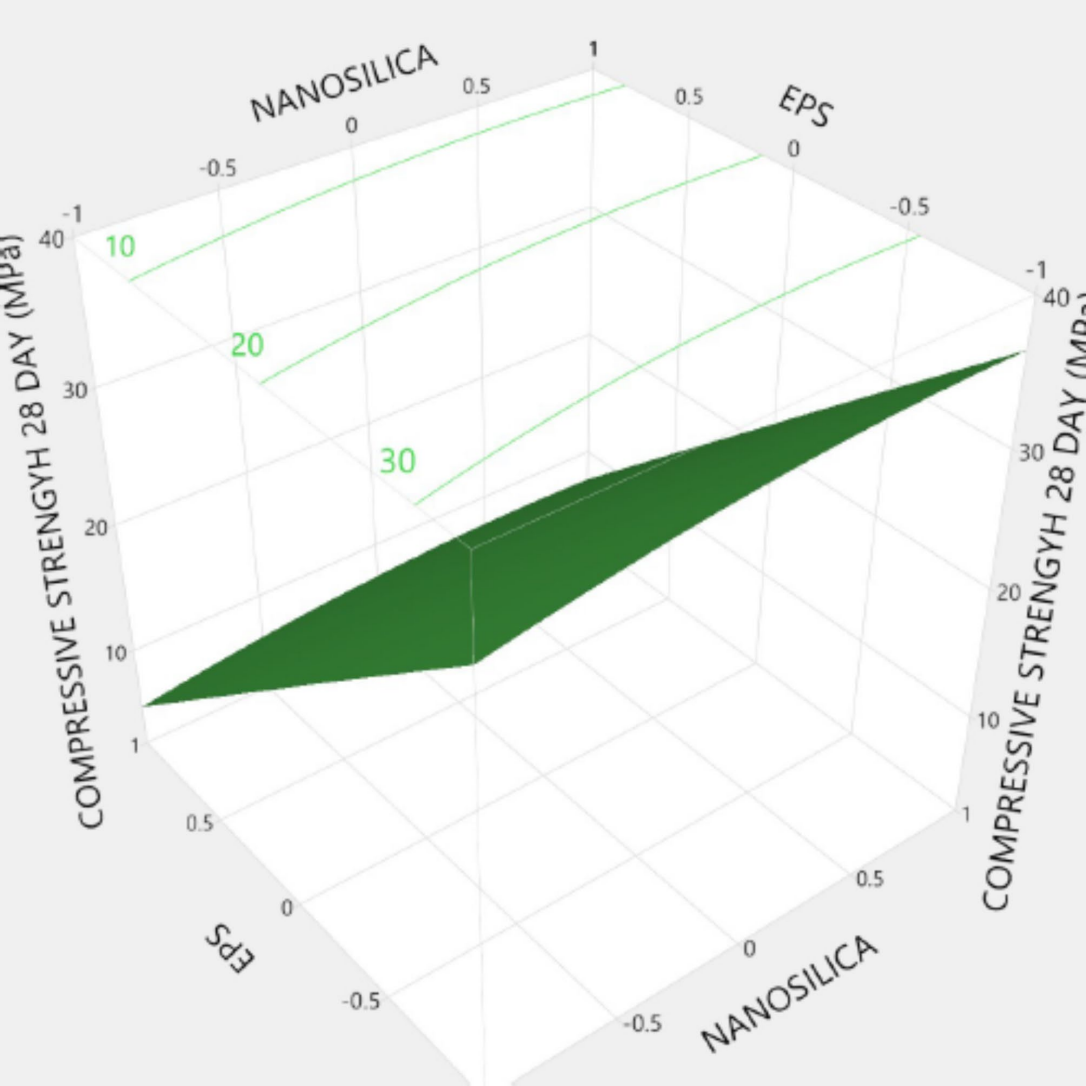
Surface Response of 28-day Compressive strength with the change, NS, and EPS percentages.

**Fig. 22:**
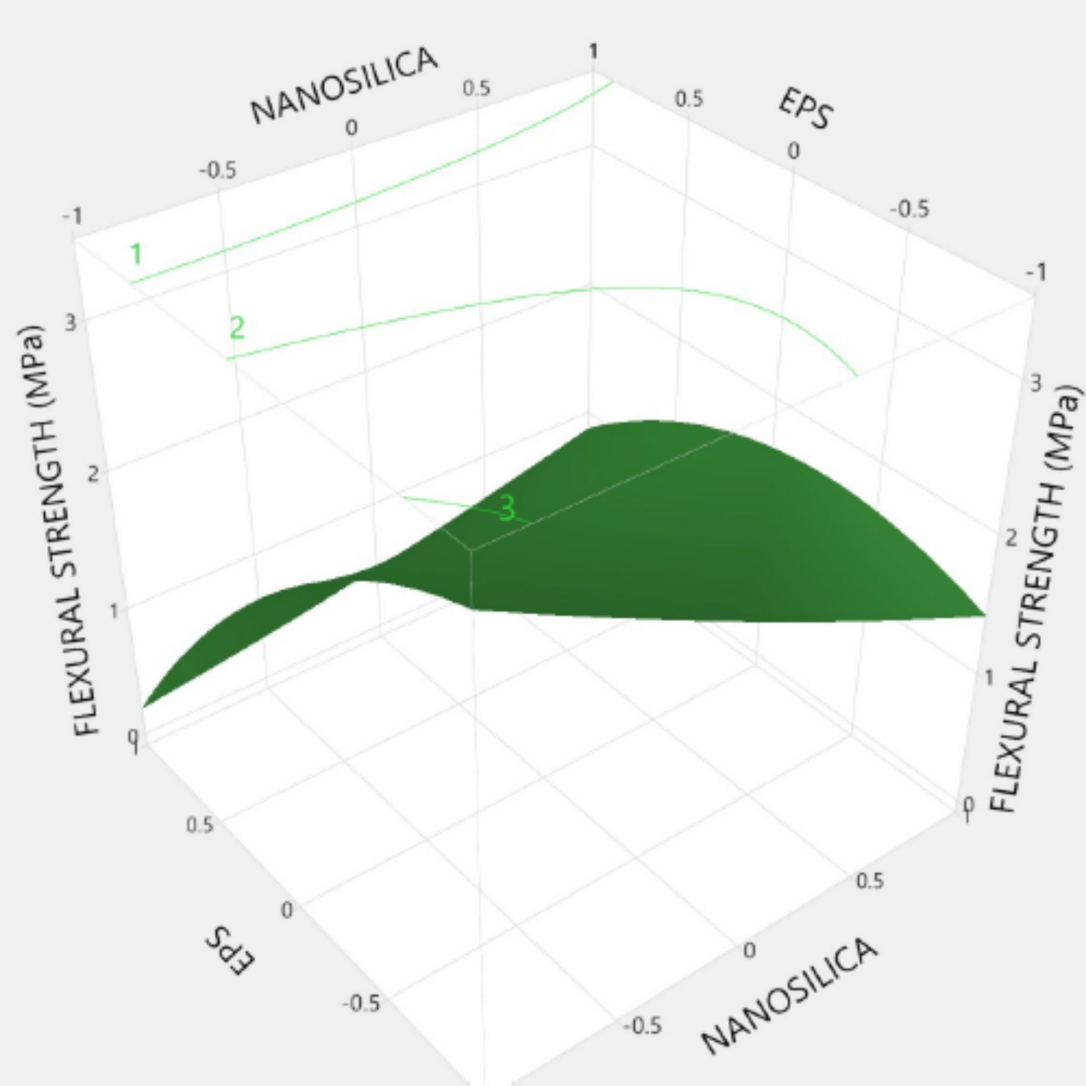
Surface Response of flexural strength with the change, NS, and EPS percentages.

**Fig. 23:**
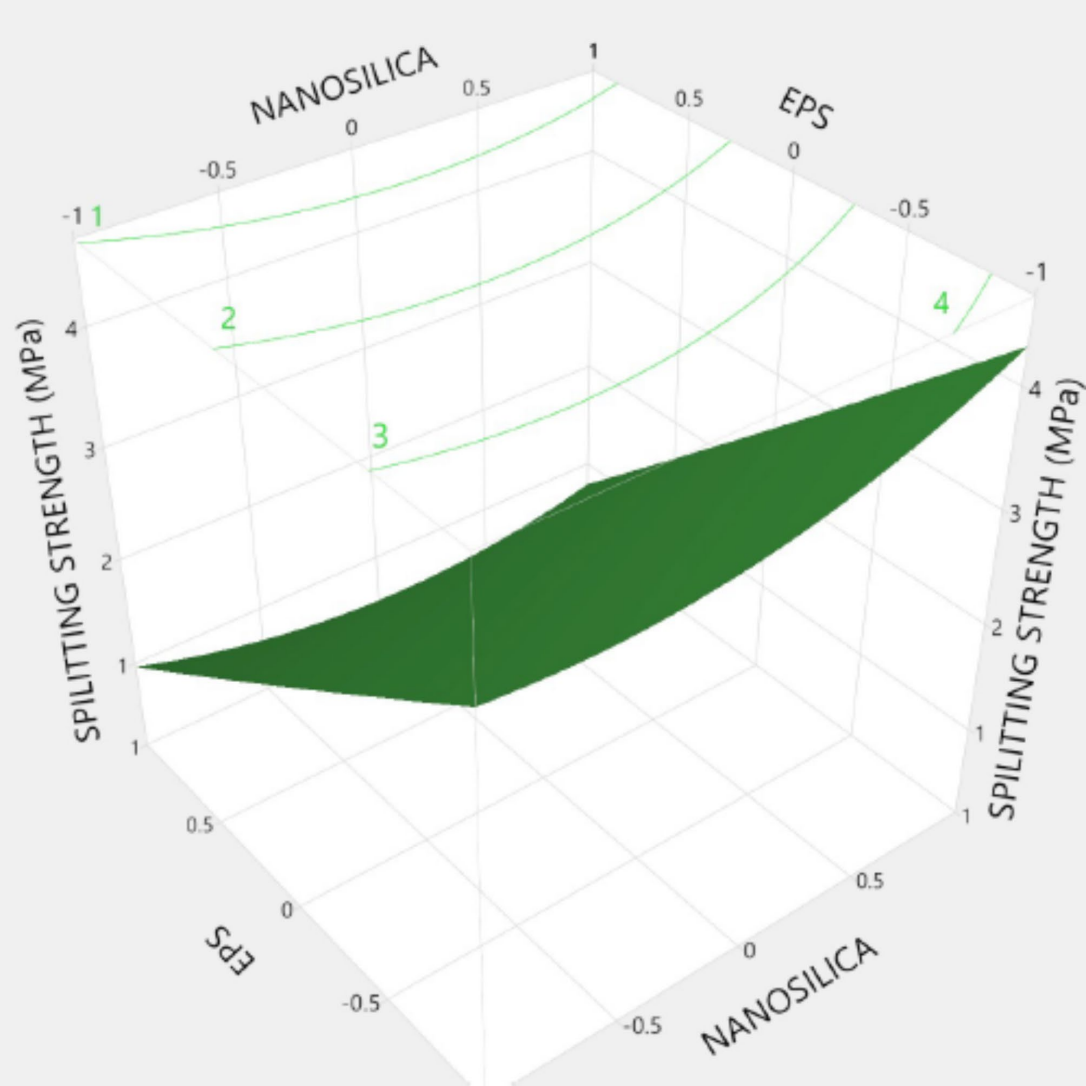
Surface Response of spilitting strength with the change, NS, and EPS percentages.

**Fig. 24:**
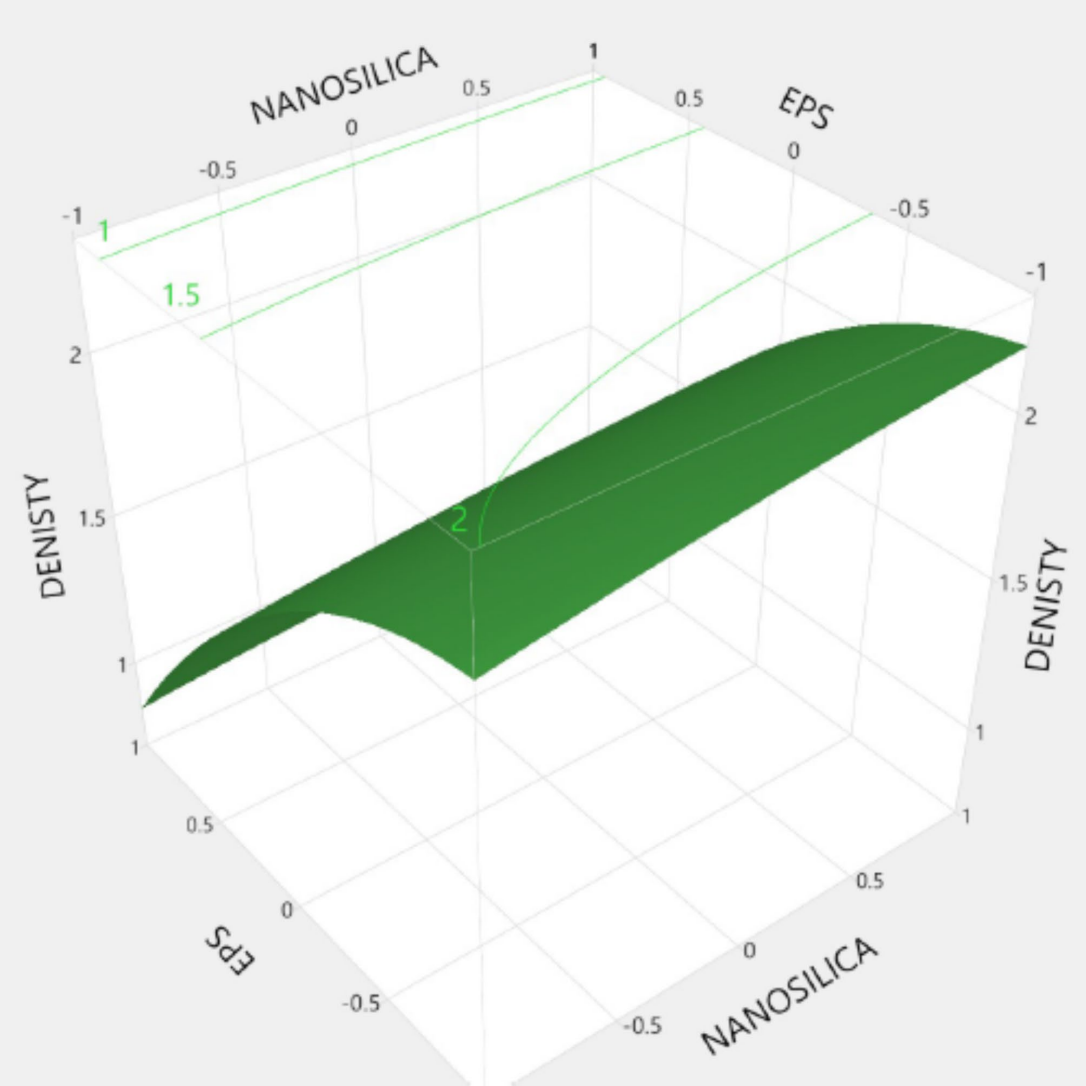
Surface Response of denisty with the change, NS, and EPS percentages.

**Fig. 25:**
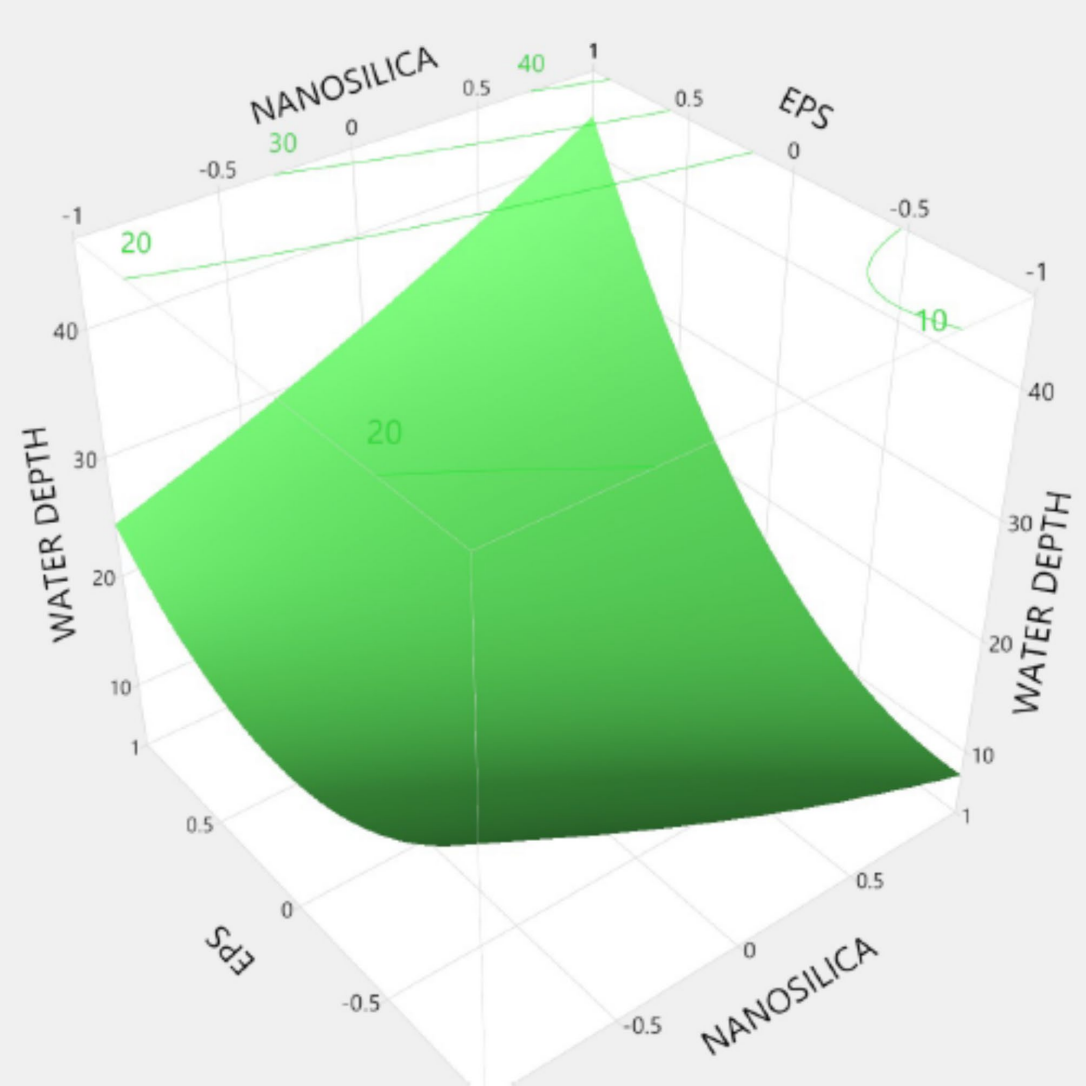
Surface Response of water depth with the change, NS, and EPS percentages.

**Fig. 26:**
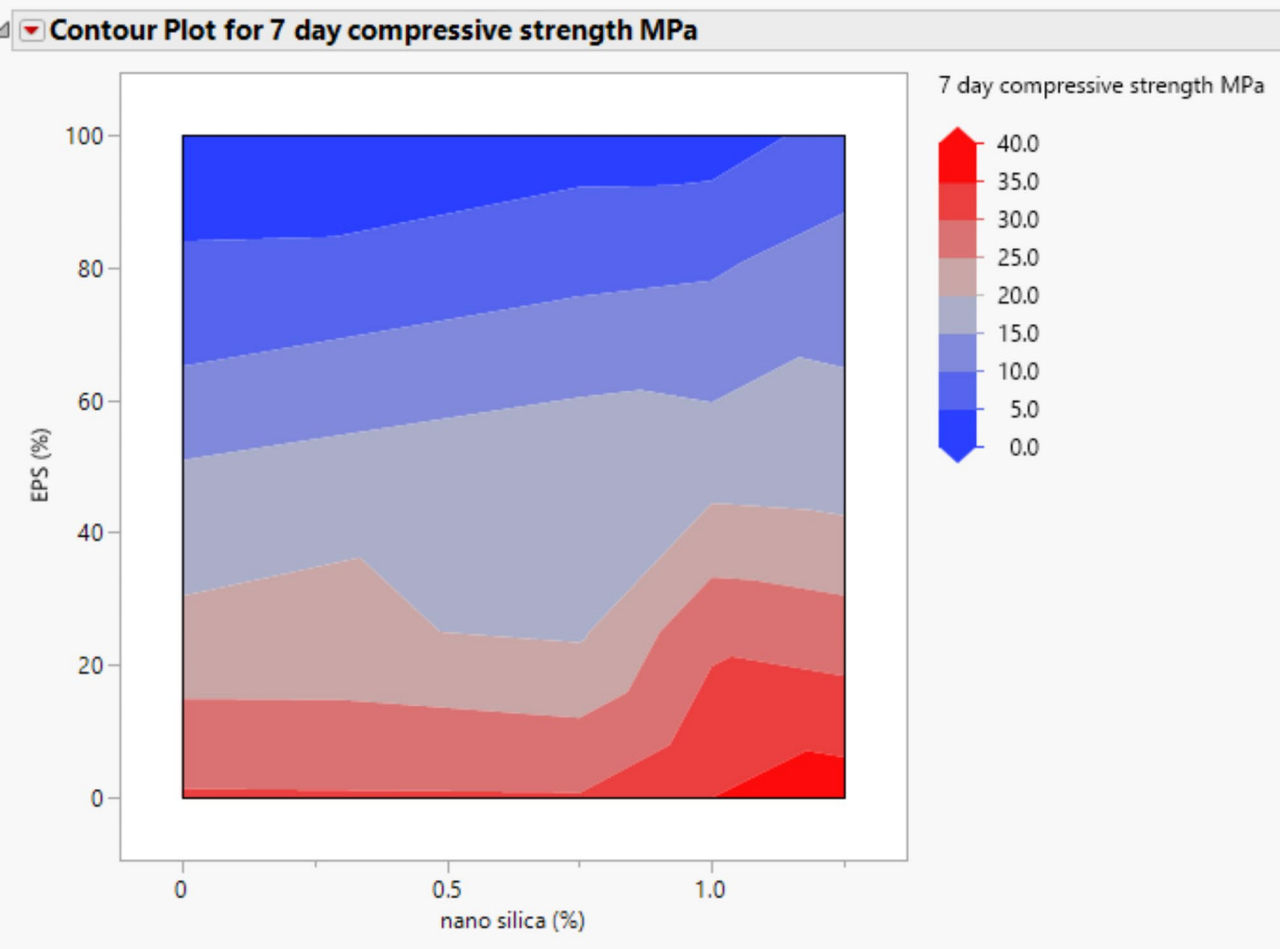
Contour plot correlating EPS, and NS with 7-daycompressive strength predicted results.

**Fig. 27:**
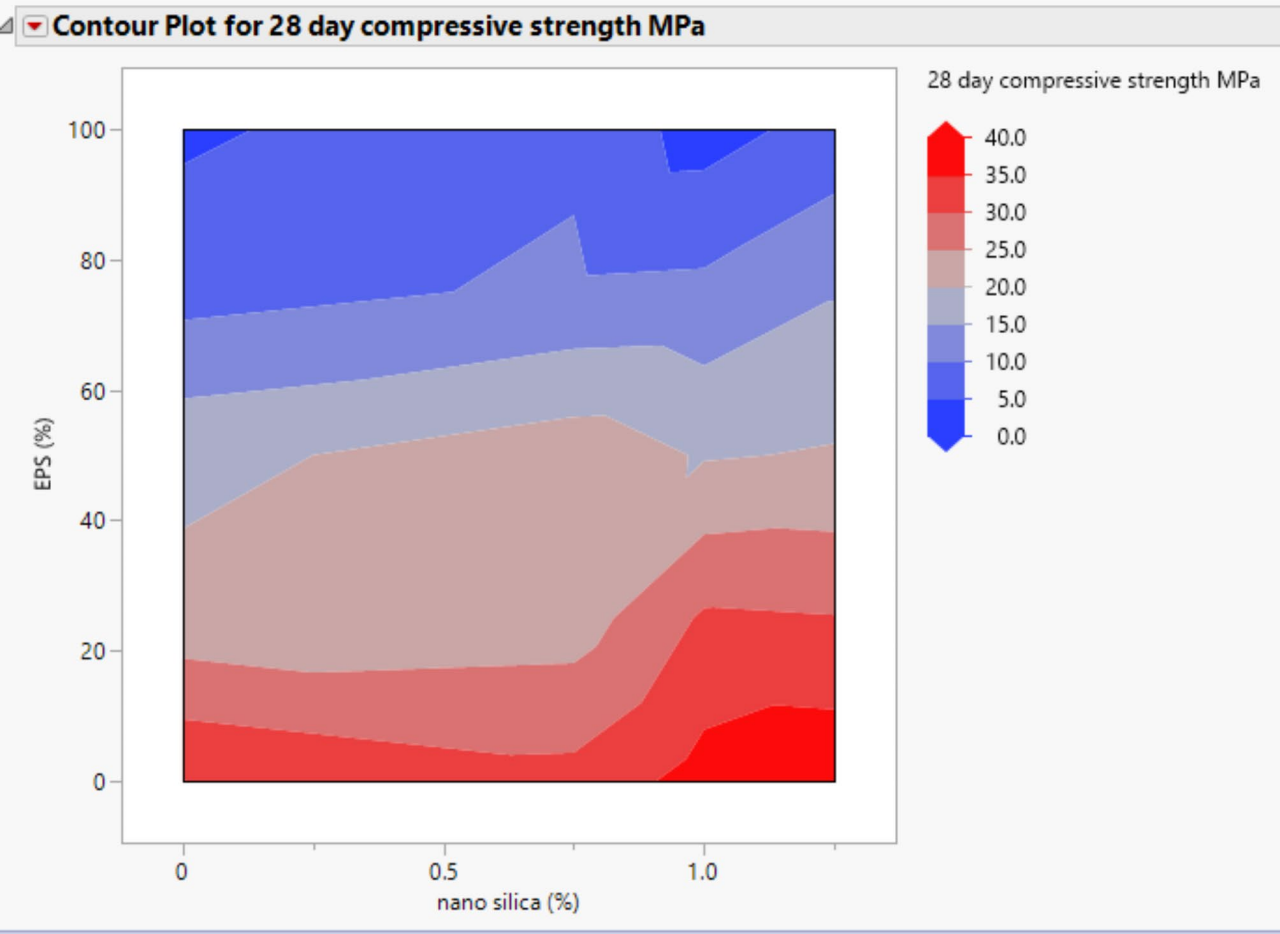
Contour plot correlating EPS, and NS with 28-daycompressive strength predicted results.

**Fig. 28:**
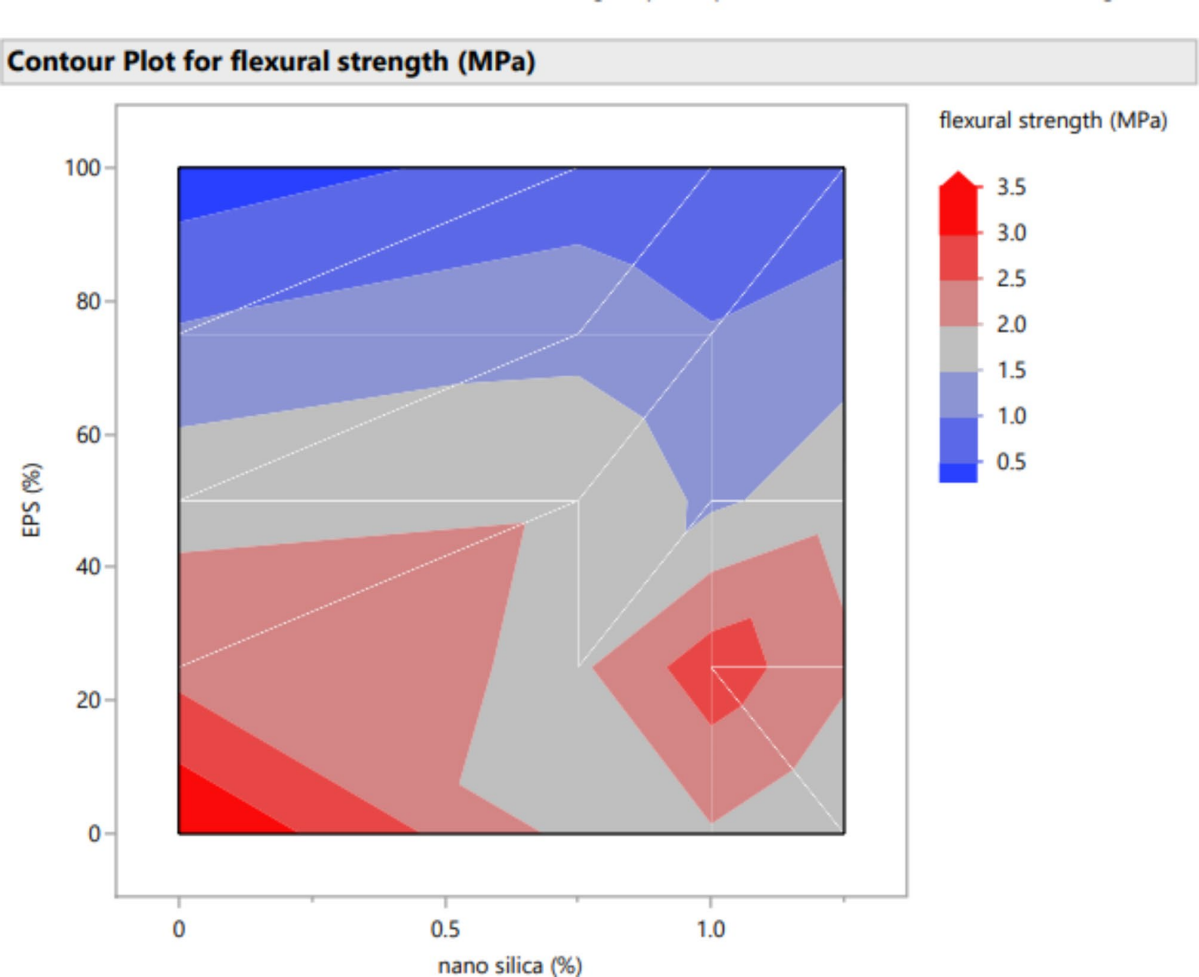
Contour plot correlating EPS, and NS with flexural strength predicted results.

**Fig. 29:**
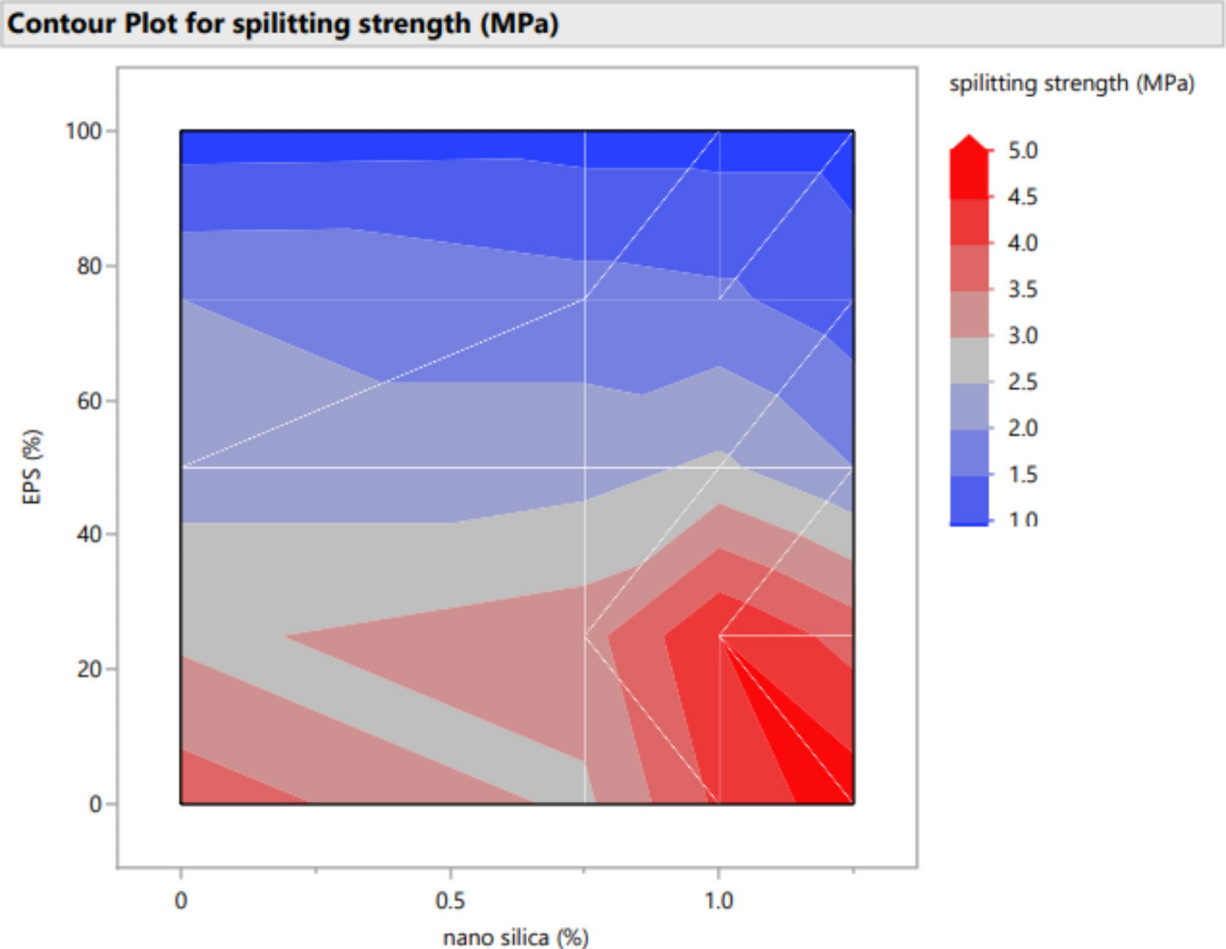
Contour plot correlating EPS, and NS with spilitting strength predicted results.

**Fig. 30:**
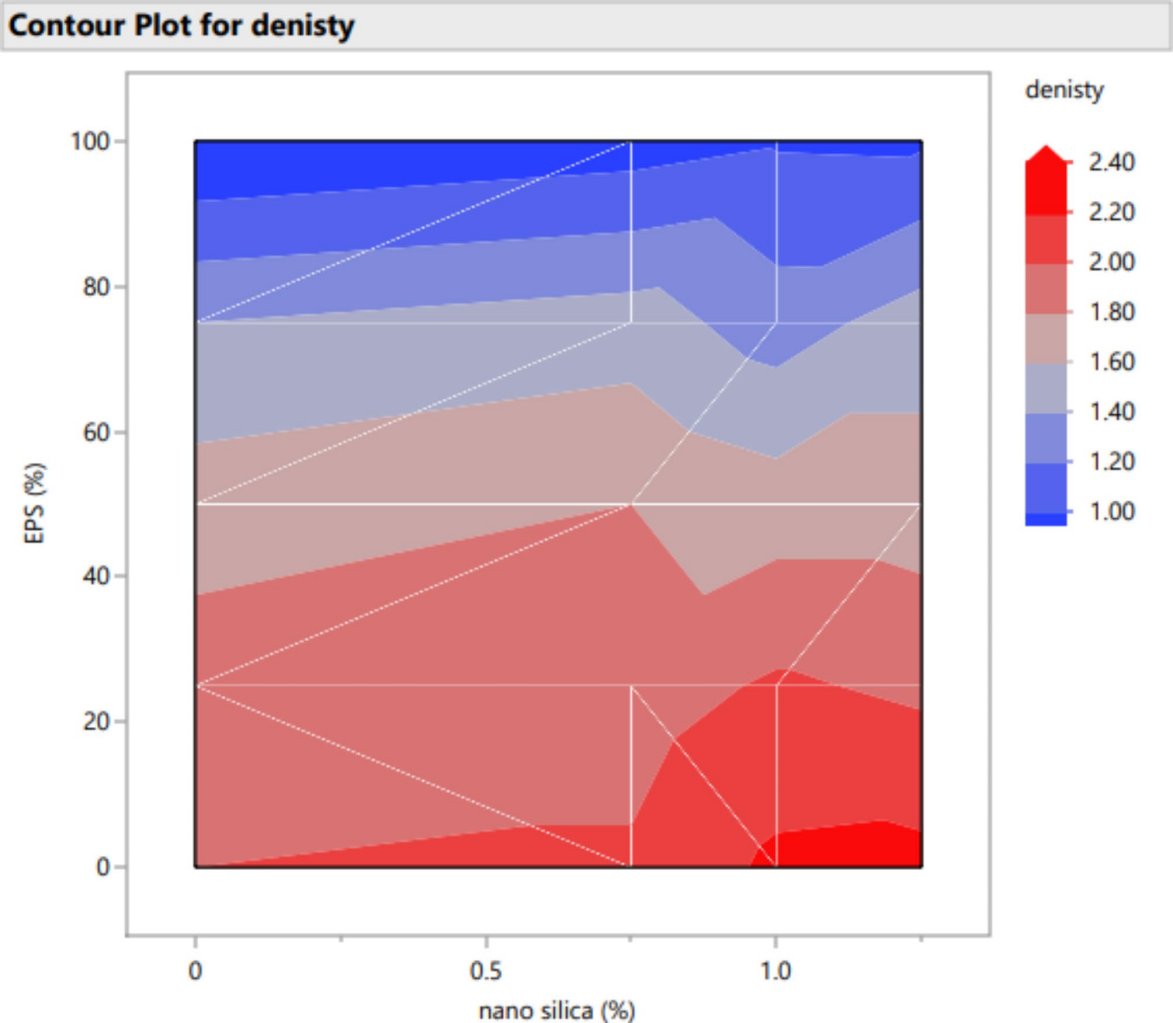
Contour plot correlating EPS, and NS with denisty predicted results.

**Fig. 31:**
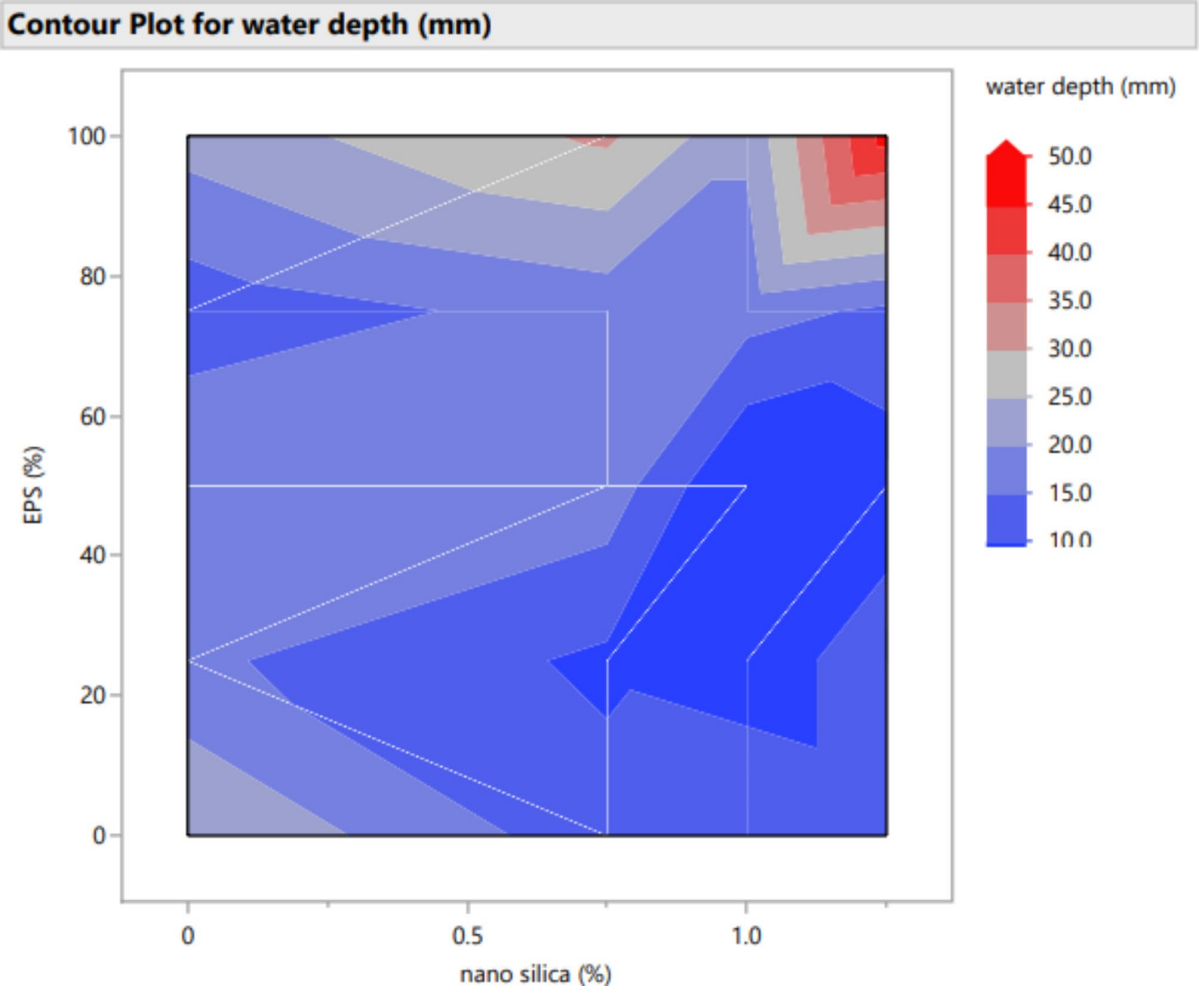
Contour plot correlating EPS, and NS with water depth predicted results.

Figures 20, 21, 22, 23, 24, 25, 26, 27, 28, 29, 30 and 31 present comprehensive design charts that establish correlations between the ratios of (EPS) and nano Silica (NS) and the predicted compressive, tensile, Flexural strength, density, water depth, and bond strengths of concrete. It is important to emphasize that the outcomes presented in these charts are derived from the specific experimental concrete mix employed in this study, thus their applicability to other concrete compositions should be carefully considered.

To optimize the performance characteristics of concrete, a thorough analysis can be conducted to determine the combination of factor levels that simultaneously fulfill the desired requirements for each response. In this simultaneous optimization process, each response is assigned a defined goal range, including options such as none, maximum, minimum, target, or within a specified range. Additionally, a weight is assigned to each goal, indicating its relative importance in the optimization process, with higher weights signifying greater importance.

Factors considered in the optimization analysis can be varied within their designated range or set at the maximum or minimum levels based on the target goal. These goals are then integrated into an overall desirability function, which reflects the desired ranges for each response. The numerical optimization process aims to achieve a desirability value close to one for each response, utilizing statistical software (in this study, JMP was utilized). By employing this approach, the highest overall desirability function can be attained, indicating an optimal combination of factor levels that meet the desired requirements for multiple responses.

The goal-seeking process initiates from a random starting point and progresses along the steepest slope towards a maximum value. It is important to note that multiple maxima may exist due to the curvature of the response surfaces and their combination within the desirability function. An ideal case is represented by a desirability value of one within the experimental domain, indicating that all response values fall within the desirable limits. Conversely, a desirability value of zero suggests that one or more responses exceed the desirable limits, indicating suboptimal performance.

**Fig. 32:**
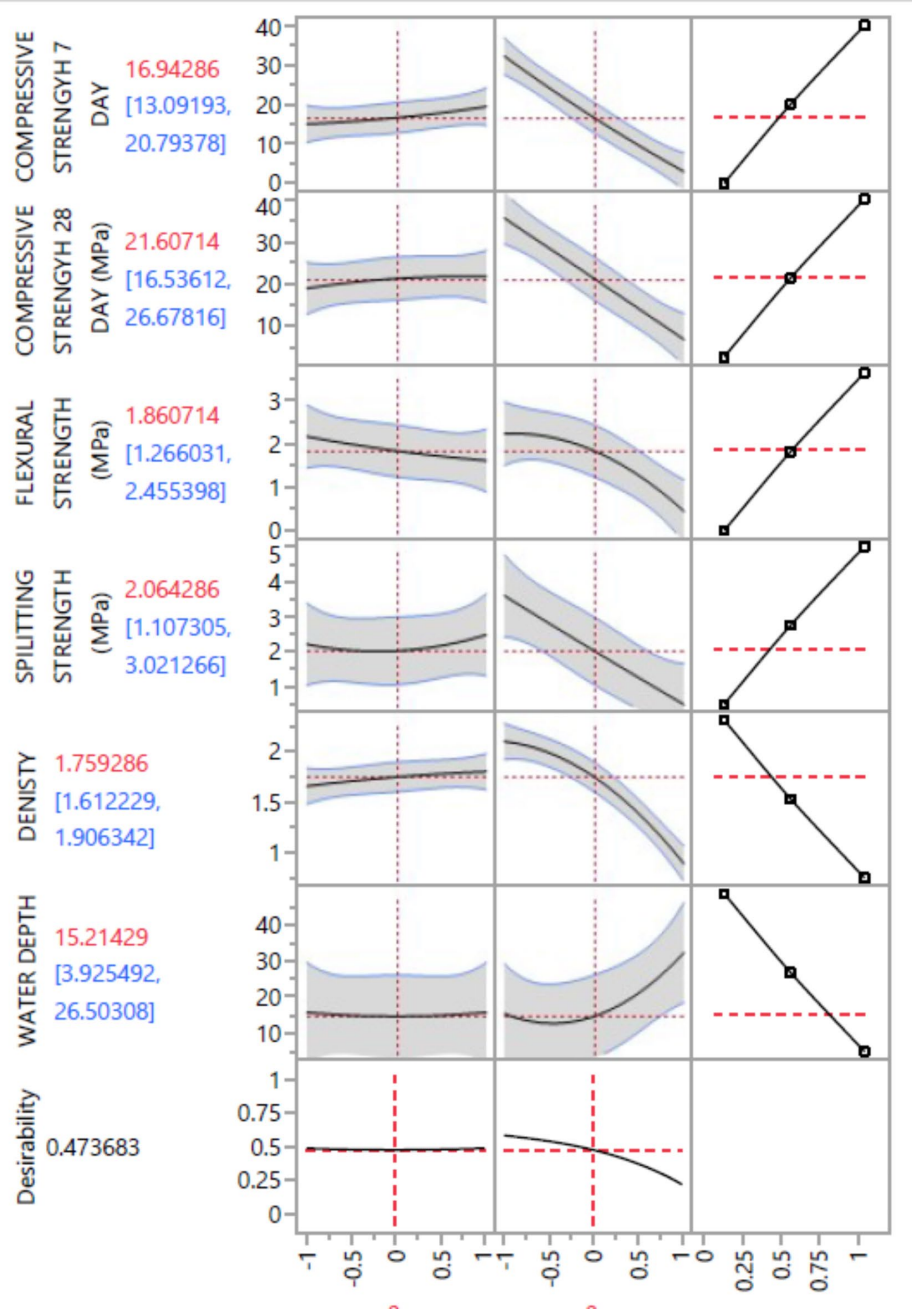
Most desirable, NS, and EPS percentages.

Finally based on the different percentage of EPS & NS the desirability percentages were introduced in Fig. 32 for all mixes as well as the corresponding predicted compressive, tensile and flexural strength density and penetration depth values.

As shown in Fig. 32, the optimum values of EPS, NS with highest desirability of 0.47 was for all mixes with a corresponding compressive, tensile and flexural strength density and penetration depth values.

Prediction expression for (compressive, tensile and flexural strength, density, penetration depth):

Based on the statistical analysis run the following prediction expression was reached to the compressive, tensile and flexural strength, density, penetration depth in terms of the replacement percentages of EPS and NS.

Prediction expression for7day compressive strength:

7 days compressive strength (MPa) = {16.94 + (2.166 * Nano silica)+ (14.4*EPS) + Nano silica* (EPS*-0.65) + Nano silica* (Nano silica* 0.614) + EPS *(EPS*0.964)}.

Prediction expression for28day compressive strength:

28 days compressive strength (MPa) = {21.6 + (1.38 * Nano silica)+ (14.2*EPS) + Nano silica* (EPS*-0.225) + Nano silica* (Nano silica* -0.914) + EPS *(EPS*0.064)}

Prediction expression for Flexural strength:

Flexural strength (MPa) = {1.86 + (0.273 * Nano silica)+ (0.88*EPS) + Nano silica* (EPS*0.6025) + Nano silica* (Nano silica* 0.053) + EPS *(EPS*-0.491)}

Prediction expression for spilitting tensile strength:

Spilitting tensile strength (MPa)= {2.06 + (0.133 * Nano silica)+ (1.53*EPS) + Nano silica* (EPS*-0.225) + Nano silica* (Nano silica* 0.32) + EPS *(EPS*0.0214)}

Prediction expression for denisty:

Denisty= {1.75 + (0.071 * Nano silica)+ (0.603*EPS) + Nano silica* (EPS*-0.0225) + Nano silica* (Nano silica* -0.018) + EPS *(EPS*-0.253)}

Prediction expression for water depth:

Water depth (mm)= {15.2 + (0 * Nano silica)+ (8.33*EPS) + Nano silica* (EPS*9.25) + Nano silica* (Nano silica* 1.071) + EPS *(EPS*9.071)}

## **Scanning electron microscopy (SEM) results**

Scanning Electron Microscopy (SEM) was employed to investigate the microstructure of lightweight concrete incorporating recycled expanded polystyrene (EPS) and the effects of nano-silica (SiO2) on its properties. The SEM images provided crucial insights into the morphology, distribution, and interaction of the concrete matrix components, including cementitious materials, aggregates, and additives.

In lightweight concrete utilizing EPS, SEM images revealed the distribution and dispersion of EPS particles within the concrete matrix, illustrating their interaction with the cementitious materials. The addition of nano-silica was assessed through SEM, which demonstrated significant microstructural changes, including the formation of denser and more compact structures in the mixtures containing nano-silica.

**Fig. 33:**
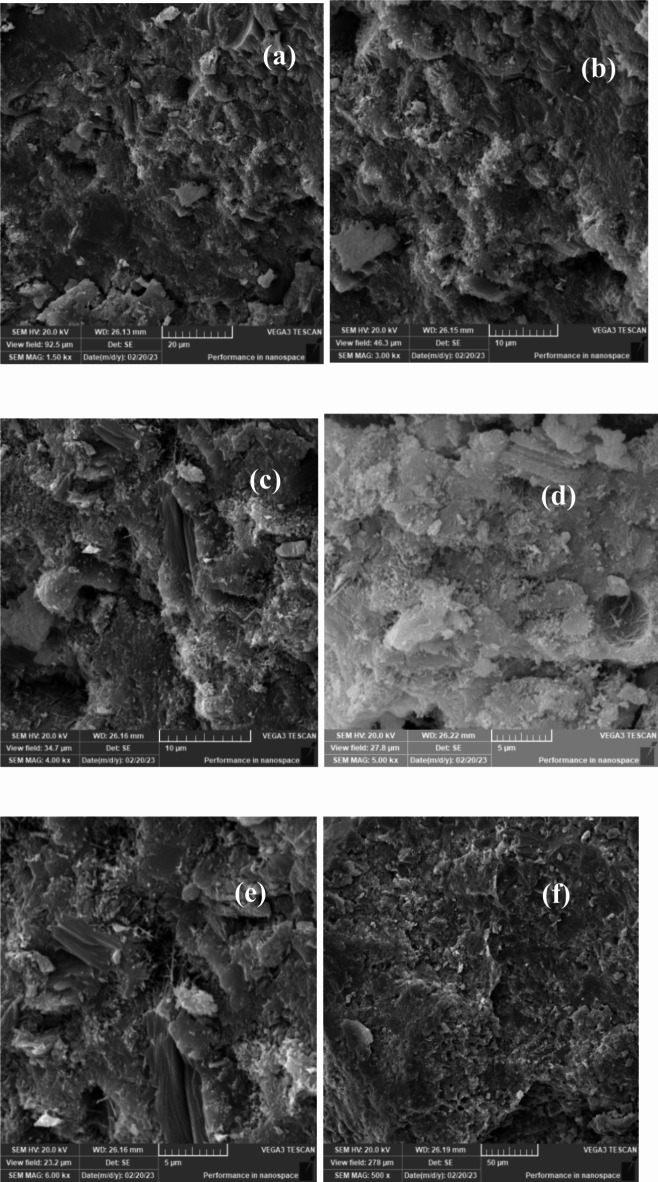
(**a**–**f**) SEM for E25( mix with25% EPS replacement without Nano silica).

**Fig. 34:**
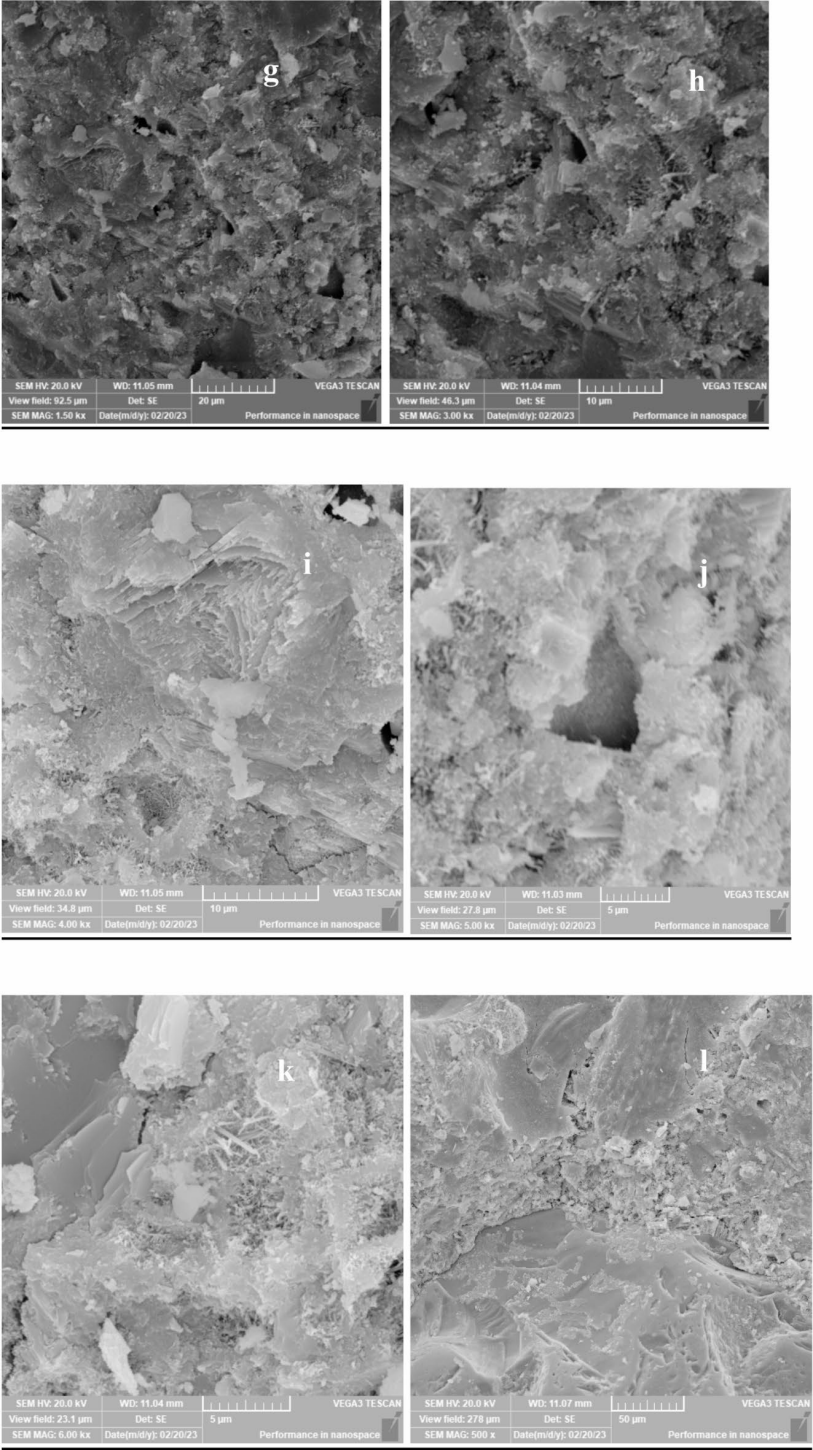
(**g**–**l**) SEM for E75( mix with75% EPS replacement without Nano silica).

**Fig. 35:**
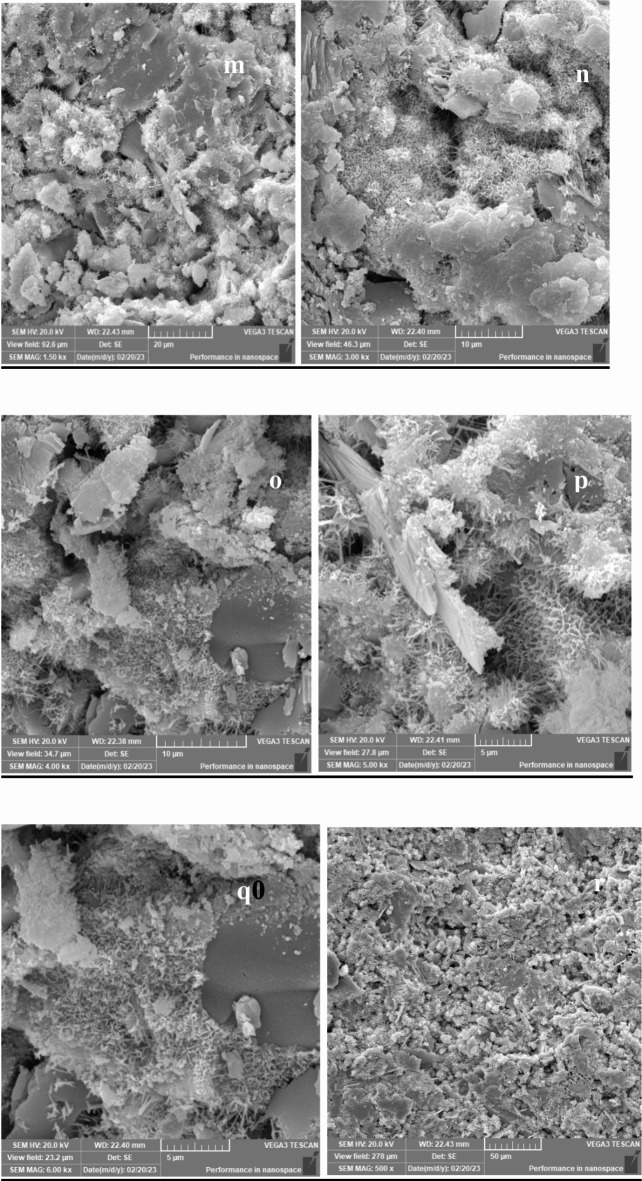
(**m**–**r**) SEM for E100 ( mix with100% EPS replacement without Nano silica).

**Fig. 36:**
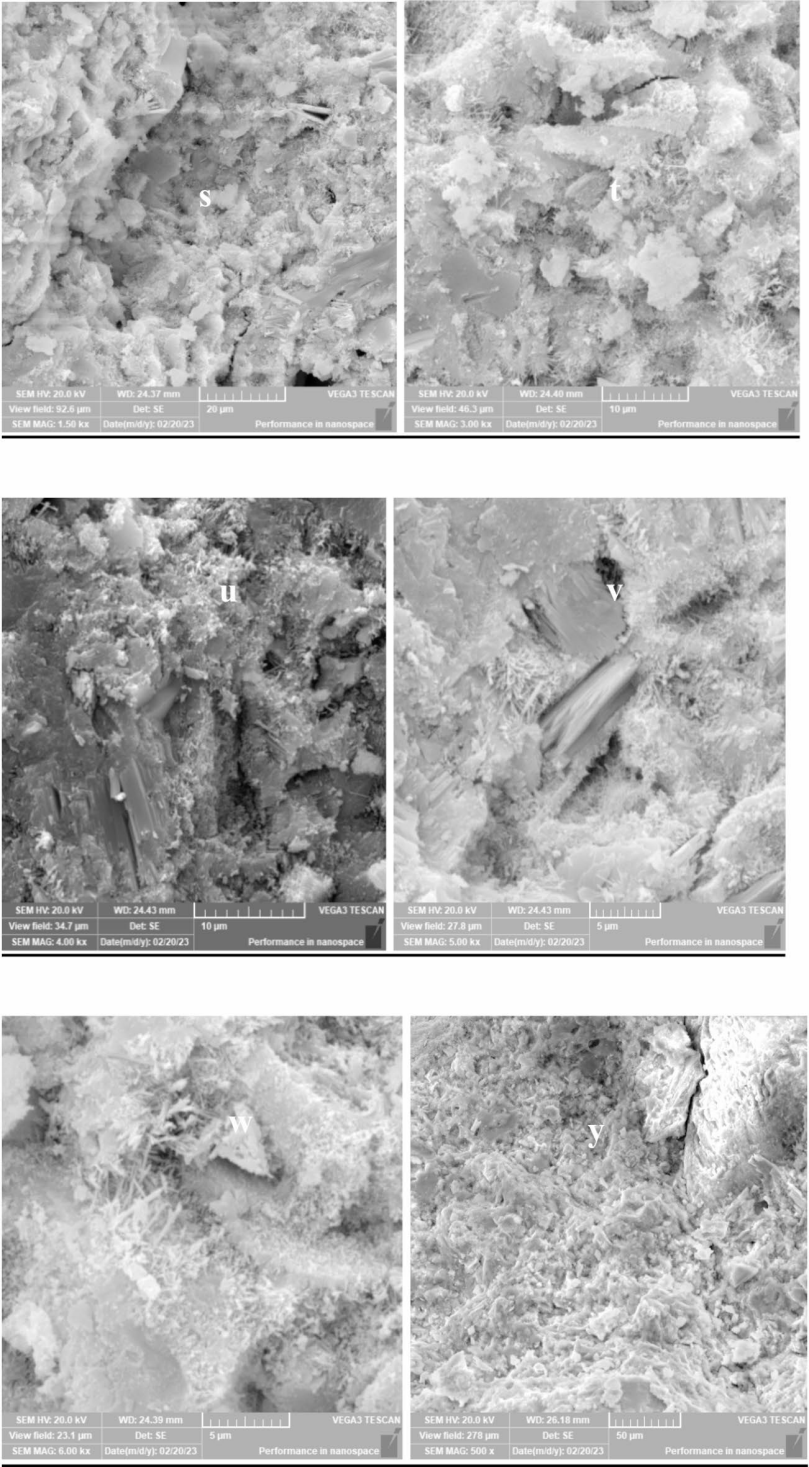
(**s**–**x**) SEM for E50N1.25 (mix with50% EPS and with1.25% Nano silica).

**Fig. 37:**
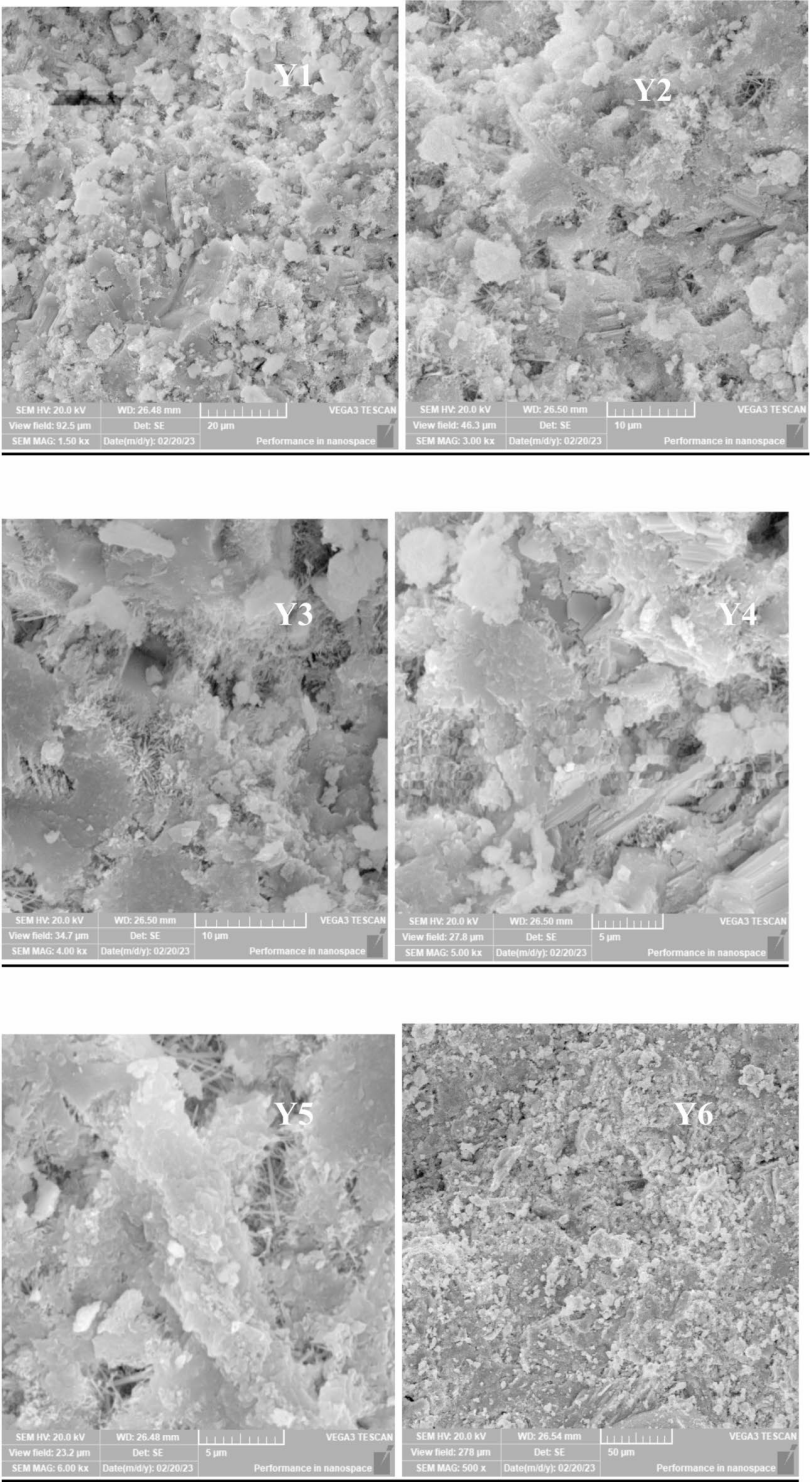
(**y1**–**y6**) SEM for E50 ( mix with50% EPS replacement without Nano silica).

Figures 33, 34, 35, 36 and 37 display SEM images of lightweight concrete using EPS with and without nano-silica additives. The incorporation of nano-silica led to a denser concrete matrix characterized by reduced porosity and enhanced interfacial bonding between EPS particles and cementitious materials. These microstructural alterations can be attributed to the filling of pores and cracks by nano-silica particles, which not only occupy voids but also promote strong chemical bonding with the cement matrix. This phenomenon is critical for improving mechanical properties, as a denser microstructure typically translates to greater load-bearing capacity.

Wang et al. (2018) reported similar findings regarding the effects of nano-silica on the microstructure of lightweight concrete using EPS, highlighting the formation of a more homogeneous and uniform matrix. The SEM images further revealed the creation of a dense interfacial transition zone between EPS particles and the cementitious materials, suggesting improved bonding that correlates with enhanced mechanical performance, particularly in compressive and flexural strength.

Moreover, detailed SEM images of the fracture surfaces at both low and high magnifications provided additional insights. The interface of samples without nano-silica appeared smooth with minimal observable fiber-shaped hydration products, indicating weaker bonding and lower mechanical performance. In contrast, samples with lower dosages of nano-silica exhibited small-sized hydration products, while those with higher dosages showcased a significant quantity of fiber-shaped hydration products at the interface. These are elongated crystalline structures that form during the hydration of cement when nano-silica is added to the mix. Their significance lies in their ability to enhance the mechanical properties of concrete. Specifically, these fiber-shaped products contribute to improved tensile strength and ductility by creating a more interconnected microstructure within the cement paste. This interlocking network helps distribute stress more evenly throughout the concrete, reducing the likelihood of cracking and enhancing overall durability. The presence of these fibers can also improve the resistance of concrete to various forms of degradation, making it suitable for demanding applications. This increase in fiber-shaped products is indicative of enhanced hydration processes, which can lead to improved tensile and flexural strengths, as observed in our mechanical testing results.

The SEM images reveal significant microstructural changes when nano-silica is incorporated into EPS-based lightweight concrete. The formation of a denser and more compact concrete matrix, as observed in the SEM images, is crucial for enhancing mechanical properties. Specifically, the presence of nano-silica facilitates the filling of voids and cracks within the concrete matrix, leading to improved interfacial bonding between EPS particles and cementitious materials. This enhanced bonding contributes to increased compressive strength, as evidenced by the substantial gains observed in our compressive strength tests, particularly in mixes with higher nano-silica contents. Furthermore, the development of fiber-shaped hydration products, particularly at higher magnifications, indicates a more effective hydration process and the formation of a robust interfacial transition zone. This zone is critical for transferring stresses between the lightweight aggregates and the cement paste, which is reflected in the improved flexural and splitting tensile strength results. By correlating the SEM observations with our mechanical property data, we can conclude that the microstructural enhancements resulting from nano-silica incorporation play a significant role in the overall performance of lightweight concrete.

## Conclusion

This study investigated the effects of nano-silica (SiO2) and recycled expanded polystyrene (EPS) on the properties of lightweight concrete. The key findings are as follows:

### Density

The incorporation of nano-silica without EPS resulted in a decrease in concrete density.

Increasing the volume of EPS consistently reduced the density of the concrete.

The combined effect of nano-silica and EPS on density was minimal, indicating that the presence of EPS predominately influences density characteristics.

### Compressive strength

The addition of nano-silica significantly enhanced compressive strength, with notable improvements observed at both 7 and 28 days.

The extent of this enhancement was dependent on the concentration of nano-silica and the percentage of EPS replacement, highlighting the need for careful mixture design.

### Flexural strength

While the addition of nano-silica generally decreased flexural strength in mixes without EPS, its impact varied with different EPS contents, suggesting a complex interaction that requires further exploration.

### Splitting tensile strength

Nano-silica improved splitting tensile strength in EPS-containing mixes, although the degree of improvement was contingent upon the specific dosages of nano-silica and EPS.

### Water permeability

The inclusion of nano-silica led to reduced water permeability, particularly at higher concentrations. However, the relationship between nano-silica and water permeability was influenced by the EPS content, indicating varying effects across different formulations.

### Microstructural insights

SEM analysis revealed that nano-silica contributed to a denser and more compact microstructure, enhancing interfacial bonding between EPS and cementitious materials. This improved bonding was linked to the observed mechanical property enhancements.

### Implications and future research

The findings underscore the potential of utilizing nano-silica to enhance the performance of lightweight concrete, particularly in applications requiring high compressive strength and reduced permeability these optimized concrete mixes could be particularly beneficial in applications such as precast concrete elements, lightweight structural components, and insulating concrete forms. The increased compressive strength and reduced permeability can lead to more durable structures with lower maintenance costs. Additionally, the lightweight nature of these mixes can reduce transportation and handling costs, making them advantageous for projects where weight is a critical factor. The intricate relationships between nano-silica dosage, EPS content, and mechanical properties suggest that optimized mixture designs could significantly improve concrete performance.

### Environmental benefits

Utilizing recycled EPS in concrete not only diverts waste from landfills but also reduces the demand for traditional aggregates, which can significantly lower the carbon footprint associated with production and transportation. Based on existing literature, it is estimated that replacing a portion of conventional aggregates with EPS can reduce the carbon footprint of concrete by approximately 30–50%. Furthermore, the use of nano-silica can enhance the efficiency of cement hydration, potentially decreasing the overall cement content required in the mix. This could lead to an additional reduction of 10–20% in carbon emissions associated with cement production. Future studies should aim to provide precise metrics on energy consumption and carbon footprint reductions for these optimized mixtures.

Future research should focus on:

- Investigating the long-term durability of lightweight concrete incorporating nano-silica and EPS.
- Exploring the optimal ratios of nano-silica and EPS to maximize mechanical properties while maintaining lightweight characteristics.
- Examining the effects of varying environmental conditions on the performance of these concrete mixtures.
- By advancing our understanding of these interactions, this research contributes to the development of sustainable construction materials and practices.

## Data Availability

The datasets used and/or analysed during the current study available from the corresponding author on reasonable request.
